# Role of the left intraparietal sulcus in the comprehension of action intensity described in sentences

**DOI:** 10.1162/IMAG.a.1371

**Published:** 2026-09-25

**Authors:** Hiroshi Shibata, Kosaku Takahashi, Mitsuki Niida, Kenji Ogawa

**Affiliations:** Department of Rehabilitation, Faculty of Medical Science and Welfare, Tohoku Bunka Gakuen University, Sendai, Japan; Department of Psychology, Graduate School of Humanities and Human Sciences, Hokkaido University, Sapporo, Japan

**Keywords:** functional magnetic resonance imaging, multivoxel pattern analysis, action intensity comprehension, left intraparietal sulcus, embodied cognition, magnitude cognition

## Abstract

From an embodied cognition perspective, semantic processing is assumed to involve sensorimotor representations. The present study used functional magnetic resonance imaging to investigate whether comprehension of action intensity described in sentences is associated with neural representations shared with action execution, particularly those related to grip force. In the execution task, participants gripped their right hand with either high or low force. In the language task, participants evaluated the action intensity described in the sentences. We examined four regions of interest (ROIs): the left primary somatosensory cortex, left primary motor cortex, left dorsal premotor cortex, and the left intraparietal sulcus (LIPS). Multivoxel pattern analysis was used to assess whether intensity information could be decoded within and across tasks. In the execution task, intensity differences were reliably decoded from multivoxel activity patterns across all four regions. In the language task, significant decoding of intensity was observed only in the LIPS. Cross-classification between the execution and language tasks did not reach significance in the primary analysis across the four regions and was associated with low effect sizes. However, exploratory analyses using smaller ROIs revealed significant cross-decoding and moderate effect sizes in the LIPS. These findings suggest that the comprehension of action intensity is associated with higher-level intensity-related representations rather than with neural representations that are closely tied to low-level motor execution. Overall, the results do not support strong embodied accounts, which assume the direct reinstatement of low-level motor execution processes during language comprehension. However, these findings suggest that partially shared higher-level representations of intensity may exist between action execution and language processing.

## 1 Introduction

In daily life, it is essential to appropriately adjust the intensity of physical movements. For example, grasping an object with excessive force may cause damage, whereas applying insufficient force may lead to dropping it. Similarly, when actions are performed based on verbal instructions such as “grip strongly” or “grip weakly,” accurate interpretation of the intensity conveyed by such expressions is important for an appropriate action execution. The present study aimed to elucidate the neural mechanisms underlying comprehension of language stimuli that describe action intensity.

The embodied cognition hypothesis posits that the comprehension of language stimuli recruits sensorimotor representations (Barsalou, 1999, 2008; Price et al., 2015; Pulvermüller, 2013). Barsalou (1999, 2008) argued that the reactivation of sensorimotor experiences, namely internal sensorimotor simulation, is involved in conceptual recognition. For example, conventional univariate analyses of brain activity have reported increased activity in the fronto-parietal regions, including the precentral gyrus, premotor cortex (PM), inferior frontal gyrus (IFG), and inferior parietal lobule (IPL) in response to action-related language stimuli (e.g., “grasp,” “kick”) (Aziz-Zadeh et al., 2006; Boulenger et al., 2009; Hauk et al., 2004; Raposo et al., 2009; Tettamanti et al., 2005; Willems et al., 2010).

However, these fronto-parietal regions have also been reported to be engaged across a variety of cognitive tasks, such as spatial and verbal working memory tasks and other domain-general processes (Fedorenko et al., 2013). Activation in these regions, by itself, is insufficient to establish that motor representations are necessarily reactivated during language comprehension. In recent years, multivoxel pattern analysis (MVPA) has been used to investigate brain activity patterns associated with action-related language comprehension and action observation. Wurm and Caramazza (2019) demonstrated that while the type of action (e.g., “give,” “take”) can be classified from activation patterns in a wide fronto-parietal region during language comprehension and action observation, only the left posterior temporal cortex (LPTC) shows cross-modal classification of action types. Similarly, Karakose-Akbiyik et al. (2023) reported that action-related events, including animate actions and inanimate object events, could be decoded across videos and sentences from activation patterns in the lateral occipitotemporal cortex (LOTC) and left inferior parietal lobule (LIPL). These MVPA studies indicate that the fronto-parietal regions represent different types of actions regardless of whether the input is a language or an observed action, whereas neural patterns shared across language and action stimuli are observed in the LOTC and LIPL. These regions have been proposed to reflect relatively abstract representations of actions rather than direct motor execution processes.

Critics have raised concerns regarding the necessity of embodied cognition in semantic comprehension (Bedny et al., 2008; Mahon, 2015; Mahon & Caramazza, 2008). Embodied cognition theories differ in the extent to which they consider simulation necessary for conceptual understanding. Even if sensorimotor representations play some role, the same representations used during actual actions or perceptions may not be strictly required for conceptual understanding. In addition, some perspectives hold that simulation itself may not be required, reflecting the broader range of views from strongly embodied to weakly embodied and disembodied approaches. As Willems and Francken (2012) emphasized, sensorimotor involvement in conceptual representations cannot be reduced to a simple binary “yes” or “no,” and a more detailed account is required.

Many studies have investigated the neural activity associated with the comprehension of language stimuli describing different types of actions. In contrast, limited studies have examined the neural activity associated with comprehension of language stimuli that describe action intensity. Some fMRI (functional magnetic resonance imaging) studies have examined the brain regions involved in processing language stimuli that vary in the level of physical effort force associated with actions (e.g., moving a picture [low effort] versus moving a sofa [high effort]) (Moody & Gennari, 2010; Urrutia et al., 2012). These studies reported that activity in the left middle frontal gyrus (LMFG), left IFG, and LIPL increased with increasing effort levels. However, these studies manipulated the level of inferred effort by varying the target objects and, thus, did not examine neural responses to language stimuli in which action intensity is explicitly conveyed through modifiers, such as adverbs (e.g., “strongly,” “weakly”). In addition, these studies focused on analyzing activation levels and did not examine the multivariate patterns underlying neural responses.

Some fMRI studies based on univariate analyses have investigated brain regions whose activity is modulated by differences in movement intensity, such as handgrip force (Cramer et al., 2002; Dai et al., 2001; Ehrsson et al., 2001; Ismail et al., 2014; Keisker et al., 2009; Spraker et al., 2007). These studies have shown that high-intensity movements increase activity in the primary motor cortex (M1) and primary somatosensory cortex (S1) compared with low-intensity movements. Other motor-related regions, including the PM, supplementary motor area, and cerebellum, also show modulation of activity depending on movement intensity, although some regions, such as the PM, do not always exhibit increases proportional to the intensity (Keisker et al., 2009). Another fMRI study examining the motor imagery of right-hand grasping at 10%, 30%, and 60% of maximum voluntary contraction (MVC) found greater activation at 60% than at 10% in the right PM, right IPL, and right S1 (Mizuguchi et al., 2014).

A recent MVPA study examined brain activity patterns during the preparation and execution of right-hand grip force that was parametrically manipulated by instruction and reported that differences in intensity could be decoded across widespread regions centered on the left M1 (LM1) and left S1 (LS1) (Caccialupi et al., 2025). This study also demonstrated that the left dorsal premotor cortex (LPMd) and left intraparietal sulcus (LIPS) exhibited cross-classification (cross-regression) of force intensity between action execution (training data) and action preparation (test data), suggesting that these regions contribute to transforming information about the intended grip force into a motor code prior to action execution. From an embodied cognitive perspective, neural processes involved in the execution of actions of varying intensity are expected to be engaged during the comprehension of language stimuli describing the action intensity. However, only a few studies have investigated language comprehension directly from this perspective.

We employed both univariate activity analyses and MVPA to investigate the brain regions involved in processing language stimuli that describe the intensity of right-hand grasping actions. Previous research has demonstrated that task demands, such as performing an action imagery task versus a semantic judgment task, can influence the observed levels of neural activity, even when identical action-related stimuli (e.g., action-related sentences) are used (Kuhnke et al., 2020; Shibata & Ogawa, 2024; Tomasino et al., 2007, 2014; van Dam et al., 2012; Willems et al., 2010; Yang & Shu, 2014). Here, this study focused specifically on the neural activity associated with the explicit conscious comprehension of action intensity conveyed by sentences, rather than on automatic or unconscious processing, using a task in which participants were required to judge the intensity of the actions. Based on the theories of embodied cognition, we hypothesized that the brain regions associated with the processing of action intensity during movement execution would also contribute to the comprehension of action intensity described in language stimuli.

Previous studies (Karakose-Akbiyik et al., 2023; Wurm & Caramazza, 2019) reported that differences in action types are represented across language and visual (action observation) modalities in the temporal and occipital cortices. In contrast, the present study focused on understanding action intensity, which led us to assume a stronger involvement of execution-related processes in language comprehension. Based on these considerations, we conducted the following analyses. Caccialupi et al. (2025) demonstrated that the force intensity of actual right-hand gripping is broadly represented, with modulation of activation levels centered on the LM1 and LS1, and that activity patterns shared across action execution and preparation are represented in the LPMd and LIPS. First, we focused on these regions of interest (ROIs) and confirmed that greater activation in the brain regions, including the LM1 and LS1, was elicited for strong-intensity grip actions and that force intensity could be reliably decoded from multivoxel activity patterns in all four regions.

Subsequently, we examined whether the representations associated with action execution were also engaged during language comprehension. We examined whether differences in the intensity of the actions described in the sentences were reflected in the activation levels and could be decoded from the multivoxel activity patterns within these ROIs during the language task itself. However, successful decoding within the language task alone would not necessarily indicate that the same neural representations are shared across execution and language conditions. Therefore, we additionally applied cross-classification MVPA to directly examine whether common neural representations contribute to both action execution and language comprehension.

From the perspective of a strong version of embodied cognition, shared neural representations between action execution and language comprehension would be expected in lower-level sensorimotor regions such as the LM1 and LS1. However, as participants did not overtly execute movements during the language task, the regions involved in motor preparation and action planning may also have contributed to the processing of action intensity. Although the LPMd and LIPS have frequently been implicated in motor preparation and action planning (Ariani et al., 2015, 2025; Caccialupi et al., 2025; Gallivan et al., 2011a, 2011b, 2013, 2016; Pilacinski et al., 2018), these fronto-parietal regions are also known to be engaged across a variety of cognitive and domain-general processes (Fedorenko et al., 2013). Therefore, successful cross-classification in these regions would not necessarily indicate the reinstatement of execution-specific motor representations. The IPS has also been implicated in the representation of magnitude information across multiple non-motor domains, such as numerical processing (Bueti & Walsh, 2009; Bulthé et al., 2014, 2015; Lyons & Beilock, 2018; Lyons et al., 2015; Pinel et al., 2004; Walsh, 2003; Wilkey et al., 2020). Accordingly, cross-decoding in LIPS may reflect more abstract representations related to action intensity or other forms of magnitude-related processing, rather than reinstatement of execution-specific motor representations.

Moreover, the IPS is functionally heterogeneous along the anterior–posterior axis, and previous studies have suggested differences in effector specificity across IPS subregions (Leoné et al., 2014; Heed et al., 2016). Recent studies have further reported that the posterior IPS encodes effector-dependent intensity information (Caccialupi et al., 2026). Accordingly, supplementary analyses using anatomically defined IPS subregions (human intraparietal areas 1–8; hIP1–hIP8) from the Jülich Probabilistic Atlas (Eickhoff et al., 2005, 2006, 2007) were conducted to examine whether intensity-related representations were localized within specific IPS subregions. We also conducted a whole-brain searchlight analysis to examine neural representations beyond the pre-defined ROIs, considering the possibility that additional brain regions may be involved in the comprehension of action intensity from language.

## 2 Methods

### 2.1 Participants

This study included 25 healthy volunteers (12 women and 13 men; mean age = 21.8 years, SD = 2.0). Handedness was assessed using the Japanese version of the FLANDERS Handedness Questionnaire, which confirmed that all participants were right handed (Nicholls et al., 2013; Okubo et al., 2014). All participants provided written informed consent before participating in the study, in accordance with the Declaration of Helsinki. This study was approved by the Ethics Committee of Hokkaido University.

Data from one participant were excluded from the analysis because the grip force for the strong condition, assessed after the fMRI session, was markedly low (outside the mean ± 2 SD range), leading to an exceptionally small difference in grip force between the strong and weak conditions. After the fMRI session, one participant reported that they had mistakenly associated the colors of the visual cues with opposite grip force intensities until the second execution block. Therefore, the data were relabeled according to the participant’s report.

### 2.2 Stimuli

In this experiment, six Japanese sentences written in kana (Japanese syllabograms) were used as stimuli, in which the grip force intensity was manipulated by adverbs (Fig. 1). The strong-grip condition included the adverbs *kataku*, *kitsuku*, and *gyutto*, which express strong intensity (e.g., “firmly,” “tightly,” “hard”). The weak-grip condition included *karuku*, *yuruku*, and *sotto*, which express weak intensity (e.g., “lightly,” “loosely,” “gently”). The stimuli were presented on a 32-inch MRI-compatible liquid crystal display (NordicNeuroLab, Bergen, Norway) with a screen resolution of 1920 × 1080 pixels. The stimulus presentation was controlled using the PsychoPy software (http://www.psychopy.org/). During fMRI scanning, participants viewed the display through a mirror mounted above the head coil. The display was positioned 170 cm away from the mirror. The longest language stimulus subtended visual angles of 8.8° horizontally and 0.7° vertically.

**Fig. 1.**
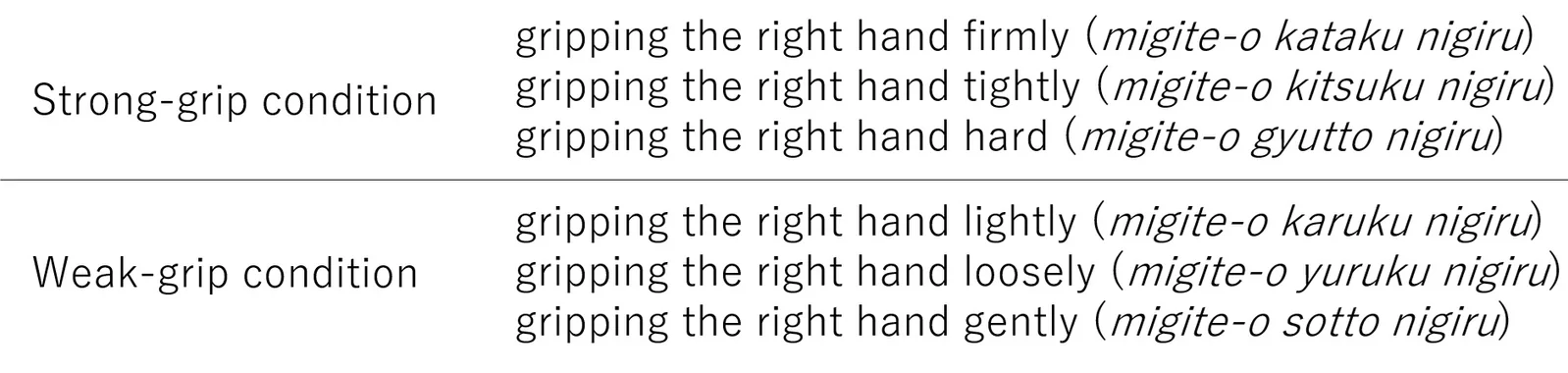
Six types of language stimuli used in this study, which were presented in Japanese.

### 2.3 Procedure

Participants performed two tasks: an execution task (Fig. 2a) and a language task (Fig. 2b). Each task included two intensity conditions, a strong-grip condition and a weak-grip condition, resulting in four experimental conditions: execution strong (ExSt), execution weak (ExWe), language strong (LaSt), and language weak (LaWe).

**Fig. 2.**
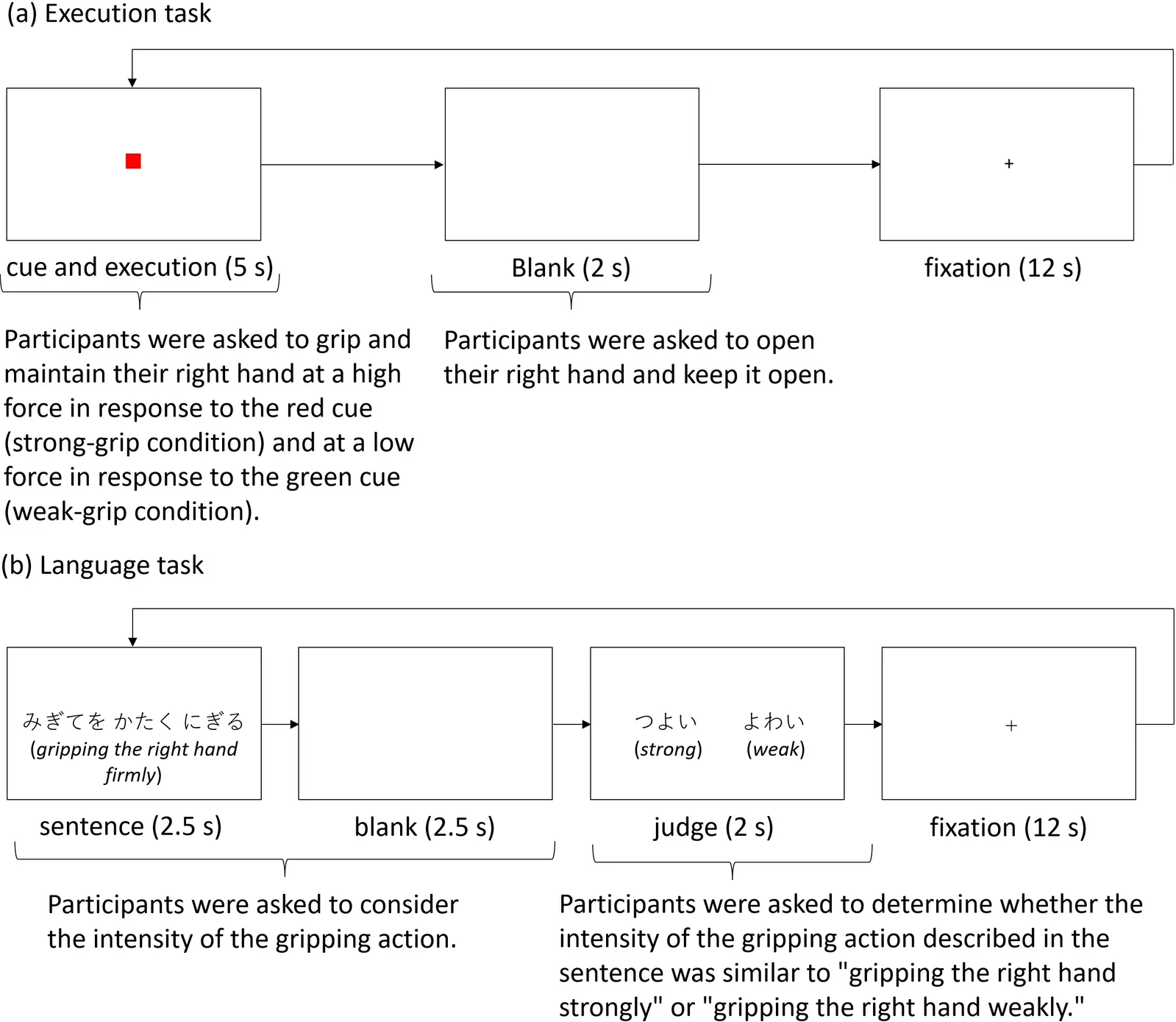
Schematic of the experimental tasks. (a) Execution task. (b) Language task.

In the execution task, participants were required to keep both palms open and facing upward (palms facing the body were also allowed), except when instructed to move their right hand. They were also asked to minimize any movements of other body parts. Before each block, participants were shown written instructions outlining the execution task, indicating that a red square corresponded to grasping with the right hand at 60% MVC, whereas a green square corresponded to grasping with the right hand at 10% MVC. Force levels of 60% and 10% were selected considering the potential fatigue from repeated right-hand gripping. After this instruction, a fixation cross was presented for 6 seconds, followed by the first trial. Each trial began with a 5-second presentation of red or green squares. Participants clenched their right hand accordingly during the action cue presentation. The order of the red and green cues was randomized, with the same-color cues restricted to occur no more than three times in a row. The cue was followed by a blank screen for 2 seconds, during which participants opened their right hand, followed by a fixation cross for 12 seconds. During fixation, participants were asked to simply gaze at the cross without any specific thoughts.

In the language task, participants were required to keep their right palm open and facing upward (or toward the body) and to place the index and middle fingers of their left hand on the response buttons of a response box while minimizing any movement of other body parts. Before each block, participants were given written instructions outlining the language task. Subsequently, a fixation cross was presented for 6 seconds, followed by the first trial. Each trial began with a 2.5-second presentation of one of the six action sentences, followed by a blank screen for 2.5 seconds. Participants were required to consider whether the presented action sentence was closer to “gripping the right hand strongly” or “gripping the right hand weakly” during the 5-second period. A 2-second response slide was then presented, during which the Japanese words for “strong (tsuyoi)” and “weak (yowai)” appeared on the left and right sides of the screen, with the positions of “strong” and “weak” counterbalanced. Participants were instructed to press the corresponding button for 2 seconds. For example, if a presented sentence was deemed as closer to “gripping the right hand strongly” and the word “strong” appeared on the left, participants pressed the left button with the middle finger of the left hand; if “strong” appeared on the right, they pressed the right button with the index finger of the left hand. The presentation order of the six sentences was randomized, with sentences of the same intensity condition restricted from occurring more than three times in a row and with the same sentence not presented consecutively.

The experiment was conducted in a single session consisting of 10 blocks, each comprising 12 trials. Five consecutive blocks were assigned to the execution task and five consecutive blocks were assigned to the language task. The order of the two tasks was counterbalanced across participants. Before fMRI scanning, participants completed practice sessions for execution and language tasks, each comprising six trials. Participants familiarized themselves with levels corresponding to 60% and 10% of their MVC before the execution task practice session. First, participants were seated on a chair with their right forearm resting on a table and palm facing upward. The participant gripped the analog dynamometer in this position and performed three maximal contractions. The median value was defined as 100% MVC. Subsequently, participants were informed of the target force levels corresponding to 60% and 10% of their MVC and were instructed to produce these forces while monitoring the dynamometer dial. Each force level was repeated thrice. The force levels were intended to provide relative differences in movement intensity rather than a precise reproduction of specific force values. Participants completed three repetitions of each force level to familiarize themselves with these relative differences rather than to train for precise force production. During the practice and experimental sessions, participants were instructed to grip their right hand at the practiced force levels in response to red and green cue stimuli. During scanning, participants’ hand movements were recorded on video from outside the MRI scanner to verify that they followed the cue stimuli, although grip force was not measured. The video recordings were reviewed after the experiment to confirm whether visible hand movements were produced during the 5-second cue period. Trials in which no visible movement was observed were treated as error trials. However, the grip force was re-measured after fMRI. Participants were instructed to grip the dynamometer at the same force levels as those produced during scanning in response to the red and green cues of the execution blocks. Participants maintained the same posture as that during the pre-experiment measurement and gripped the dynamometer without visual feedback. Each force was assessed in triplicate.

### 2.4 fMRI data acquisition

A 3-T Prisma scanner (Siemens, Erlangen, Germany) with a 64-channel head coil was used to acquire T2*-weighted echo-planar images. A total of 78 scans were obtained for each run using a gradient echo-planar imaging sequence. The scanning parameters were as follows: 3000 ms repetition time (TR), 30 ms echo time (TE), 90° flip angle (FA), 192 × 192 mm field of view (FOV), 94 × 94 matrix, 36 axial slices, and 3.0 mm slice thickness with a 0.75 mm gap. T1-weighted anatomical image was acquired using the following parameters: 2300 ms TR, 2.32 ms TE, 8° FA, FOV 256 × 256 mm, matrix 256 × 256, 192 axial slices, and 1 mm slice thickness without a gap.

### 2.5 fMRI data analysis

#### 2.5.1 Univariate activity analysis

Image preprocessing and statistical analyses were performed using the SPM12 software (http://www.fil.ion.ucl.ac.uk/spm/software/spm12/). The first two scans of each run were discarded to allow for T1 equilibration. All functional images were realigned to correct for movement artifacts, co-registered to the participant’s anatomical T1-weighted image, spatially normalized to the Montreal Neurological Institute (MNI) template with a 3 × 3 × 3 mm voxel size, and smoothed using a 6 × 6 × 6 mm Gaussian kernel. Condition effects at each voxel were estimated according to the general linear model. The 5-second periods of action cue presentation in the execution task and sentence and following blank presentation in the language task, excluding trials with no responses and incorrect responses, were modeled as separate box-car functions convolved with a canonical hemodynamic response function. The six realignment parameters were included as regressors to correct for signal changes due to head movement. Block effects were included to account for nuisance covariates. A high-pass filter with a 128-second cut-off period was applied to remove low-frequency noise. Whole-brain analyses were performed using one-sample t-tests. Activation clusters were considered significant if they exceeded a voxel-wise threshold of *p* < 0.001 (uncorrected) and a cluster-wise threshold of *p* < 0.05, family-wise error (FWE) corrected for multiple comparisons across the whole brain. The ROIs were defined based on Caccialupi et al. (2025) as LS1 (MNI: x = -32, y = -34, z = 58), LM1 (MNI: x = -43, y = -14, z = 48), LPMd (MNI: x = -26, y = -10, z = 58), and LIPS (MNI: x = -30, y = -54, z = 52), each with a 12-mm spherical radius.

#### 2.5.2 MVPA

The multivariate pattern classification analysis of the fMRI data was performed using a linear support vector machine implemented in LIBSVM (https://www.csie.ntu.edu.tw/~cjlin/libsvm/) with default parameters (fixed regularization parameter C = 1). The first-level GLM for the MVPA was identical to that described above, except that it was estimated on unsmoothed normalized images, to avoid blurring the fine-grained information carried by multivoxel activity patterns (Misaki et al., 2013; Mur et al., 2009). Parameter estimates (β values) for each trial from all voxels within each ROI were used as inputs to the classifier. The ROIs were the same as the four regions defined in the univariate activity analysis. First, for the execution trials, the decoder was trained to classify the two conditions (strong vs. weak). Next, for the language trials, the decoder was likewise trained to classify the two conditions (strong vs. weak). In this analysis, the average decoding accuracy was estimated using a five-fold leave-one-out cross-validation across five blocks, with four blocks used for training and the remaining block used for testing. Cross-decoding between execution and language trials was conducted in three analyses: a direction-averaged analysis as the primary analysis, and two directional analyses (execution-to-language and language-to-execution) as supplementary analyses. To equalize the number of trials between the strong and the weak conditions, we randomly selected an equal number of trials from each condition (downsampling). Downsampling was performed independently for each decoding analysis. For cross-decoding, the classifier was trained on data from all five blocks of one task and tested on data from all five blocks of the other task to classify the two conditions (strong vs. weak).

One-sample, one-sided *t*-tests were conducted to test whether the decoding accuracy was significantly above chance (50%), with the effect sizes (Cohen’s *d*) and Bayes factors reported for each ROI. Bayes factors were computed using JASP (version 0.96) to complement conventional significance testing. To evaluate the sensitivity of the *t*-test given the final sample size (*n* = 24) and α = 0.05, a sensitivity power analysis was conducted, indicating that an effect size of *d* = 0.523 could be detected with 80% power. To assess the robustness, we additionally tested 10-mm and 15-mm spherical ROIs. Furthermore, because the IPS extends along the anterior–posterior axis and may not be optimally captured by spherical ROIs, supplementary analyses were conducted using anatomically defined LIPS subregions (left hIP1–hIP8) from the Anatomy Toolbox (Version 3.0). Correction for multiple comparisons was applied separately to the four main functional ROIs and to the eight anatomical ROIs using the Bonferroni method.

As control analyses, we conducted two additional analyses to assess the robustness of the main results and potential confounding factors. First, after the experiment, we observed that the grip force in the weak condition differed significantly between the pre- and post-experiment measurements. To assess how this deviation affected the decoding results, supplementary ROI decoding analyses were performed after excluding five participants who showed large pre–post deviations in the weak condition (more than a three-fold increase from pre to post). In addition, a correlation analysis was conducted between the pre–post deviation in the weak movement condition and LIPS classification accuracy.

Second, we examined the potential carryover or memory effects related to the task order. Participants were divided into two groups according to task order (execution-first vs. language-first). This comparison tested whether prior experience with one task biased performance or neural decoding in the subsequent task (e.g., covert verbalization or motor imagery). Decoding accuracies were compared between groups using two-sample *t*-tests for each ROI and for each of the three main decoding analyses (execution, language, and direction-averaged cross-decoding).

We conducted a volume-based searchlight analysis to complement the a priori ROI analysis (Kriegeskorte et al., 2006). Classification was performed using multivoxel activation patterns within spherical searchlights with a radius of 12 mm. These searchlights were moved throughout the gray matter of the whole brain. Because spatial normalization was performed during preprocessing prior to model estimation, the searchlight accuracy maps were generated in the normalized MNI space and were averaged across participants without any further spatial transformation. In addition, to assess the robustness of the results at a smaller spatial scale, supplementary searchlight analyses (execution, language, and three cross-decoding analyses) were performed using a 9-mm radius. The cross-validated classification accuracy was assigned to the center voxel for each searchlight. Individual accuracy maps were then averaged across participants. Statistical maps were thresholded at a voxel-wise uncorrected *p* < 0.001 and a cluster-level FWE corrected *p* < 0.05 for each type of classification analysis. Supplementary analyses are provided showing searchlight results for the language condition and cross-decoding (direction-averaged) condition at a more lenient voxel-wise threshold of *p* < 0.05 (uncorrected) to facilitate visualization of subthreshold patterns. Brain images illustrating the results of the univariate activity analyses and MVPA were created using the MRIcroGL software (https://www.nitrc.org/projects/mricrogl).

## 3 Results

### 3.1 Behavioral results

The right-hand grip forces measured before and after the experimental sessions (pre: with visual guidance; post: without visual feedback) had the following means (standard deviations; *SD*s): pre-experiment 60% condition, 11.5 kg (3.4); pre-experiment 10% condition, 1.9 kg (0.6); post-experiment 60% condition, 11.3 kg (4.0); post-experiment 10% condition, 4.2 kg (2.0). A two-way ANOVA revealed significant main effects for measurement phase (pre vs. post), *F*(1, 23) = 4.65, *p* < 0.05, η_p_² = 0.17, and force intensity level (60% vs. 10%), *F*(1, 23) = 298.02, *p* < 0.001, η_p_² = 0.93, as well as a significant interaction between the two factors, *F*(1, 23) = 20.16, *p* < 0.001, η_p_² = 0.47. Post hoc comparisons indicated a significant difference between the 60% and 10% conditions in the pre- and post-experiment measurements (pre: *F*(1, 23) = 248.79, *p* < 0.001, η_p_² = 0.92; post: *F*(1, 23) = 199.81, *p* < 0.001, η_p_² = 0.90). In addition, the 10% condition showed a significant difference between the pre- and post-experiment measurements (60%: *F*(1, 23) = 0.08, ns, η_p_² = 0.00; 10%: *F*(1, 23) = 40.51, *p* < 0.001, η_p_² = 0.64). These results confirmed a significant difference in the right-hand grip force between the 60% and 10% conditions, although the 10% condition showed a significant change in the grip force between the pre- and post-experiment measurements. The mean percentages (*SD*s) of nonresponse trials in the execution blocks and incorrect or nonresponse trials in the language blocks were as follows: ExSt, 0.6% (1.6); ExWe, 0.7% (1.7); LaSt, 4.7% (6.1); and LaWe, 5.1% (7.5), indicating that participants performed at a high level of accuracy.

### 3.2 Univariate analysis of activation levels

The results of the univariate activity analyses are shown in Figure 3 and Supplementary Table S1. The contrast of ExSt compared with ExWe revealed greater activation centered in the left precentral and postcentral gyri, extending to the supplementary motor area, as well as the subcortical and cerebellar regions including the bilateral thalamus, basal ganglia (left putamen, left globus pallidus, and bilateral caudate nucleus), bilateral cerebellum, and bilateral hippocampus. No significant activation was observed in the contrasts of ExWe compared with ExSt, LaSt compared with LaWe, or LaWe compared with LaSt.

**Fig. 3.**
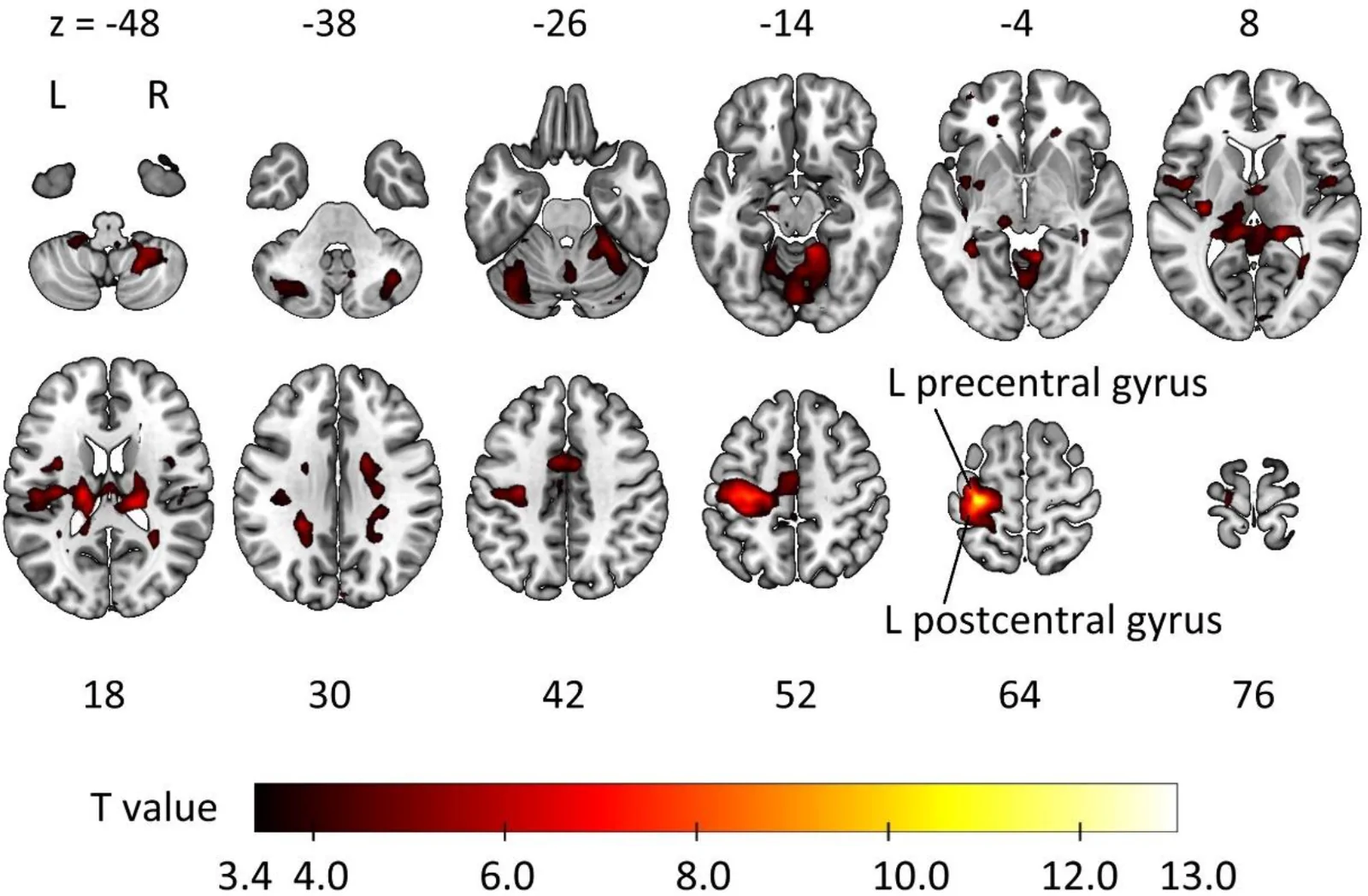
Brain regions revealed by the contrast between strong- and weak-grip conditions in the execution task using the univariate activity analysis. Results were thresholded at *p* < 0.001 uncorrected at the voxel level and *p* < 0.05 FWE corrected at the cluster level. L = Left; R = Right.

Figure 4 shows the parameter estimates (beta values) for each condition in the four functional ROIs. A two-way ANOVA with task (execution vs. language) and intensity (strong vs. weak) factors was performed for each ROI. The results revealed significant main effects of task (LS1: *F*(1, 23) = 69.67, *p* < 0.001, η_p_² = 0.75; LM1: *F*(1, 23) = 47.92, *p* < 0.001, η_p_² = 0.68; LPMd: *F*(1, 23) = 25.06, *p* < 0.001, η_p_² = 0.52) and intensity (LS1: *F*(1, 23) = 53.04, *p* < 0.001, η_p_² = 0.70; LM1: *F*(1, 23) = 27.10, *p* < 0.001, η_p_² = 0.54; LPMd: *F*(1, 23) = 10.90, *p* < 0.005, η_p_² = 0.32), and significant interactions (LS1: *F*(1, 23) = 56.09, *p* < 0.001, η_p_² = 0.71; LM1: *F*(1, 23) = 26.10, *p* < 0.001, η_p_² = 0.53; LPMd: *F*(1, 23) = 9.86, *p* < 0.005, η_p_² = 0.30) in the LS1, LM1, and LPMd. Post hoc comparisons revealed that in all three regions, the beta value in ExSt was significantly greater than that in ExWe (LS1: *F*(1, 23) = 68.72, *p* < 0.001, η_p_² = 0.75; LM1: *F*(1, 23) = 35.47, *p* < 0.001, η_p_² = 0.61; LPMd: *F*(1, 23) = 15.99, *p* < 0.001, η_p_² = 0.41), whereas no significant difference was observed between LaSt and LaWe (LS1: *F*(1, 23) = 0.00, ns, η_p_² = 0.00; LM1: *F*(1, 23) = 0.01, ns, η_p_² = 0.00; LPMd: *F*(1, 23) = 0.13, ns, η_p_² = 0.01). For reference, the comparisons between ExSt and LaSt and between ExWe and LaWe were significant in all three regions. In the LIPS, a significant main effect of the task was observed (*F*(1, 23) = 10.88, *p* < 0.005, η_p_² = 0.32), with greater activity during the language task than during the execution task. However, no significant main effect of intensity (*F*(1, 23) = 0.62, ns, η_p_² = 0.03) or interaction (*F*(1, 23) = 0.33, ns, η_p_² = 0.01) was observed.

**Fig. 4.**
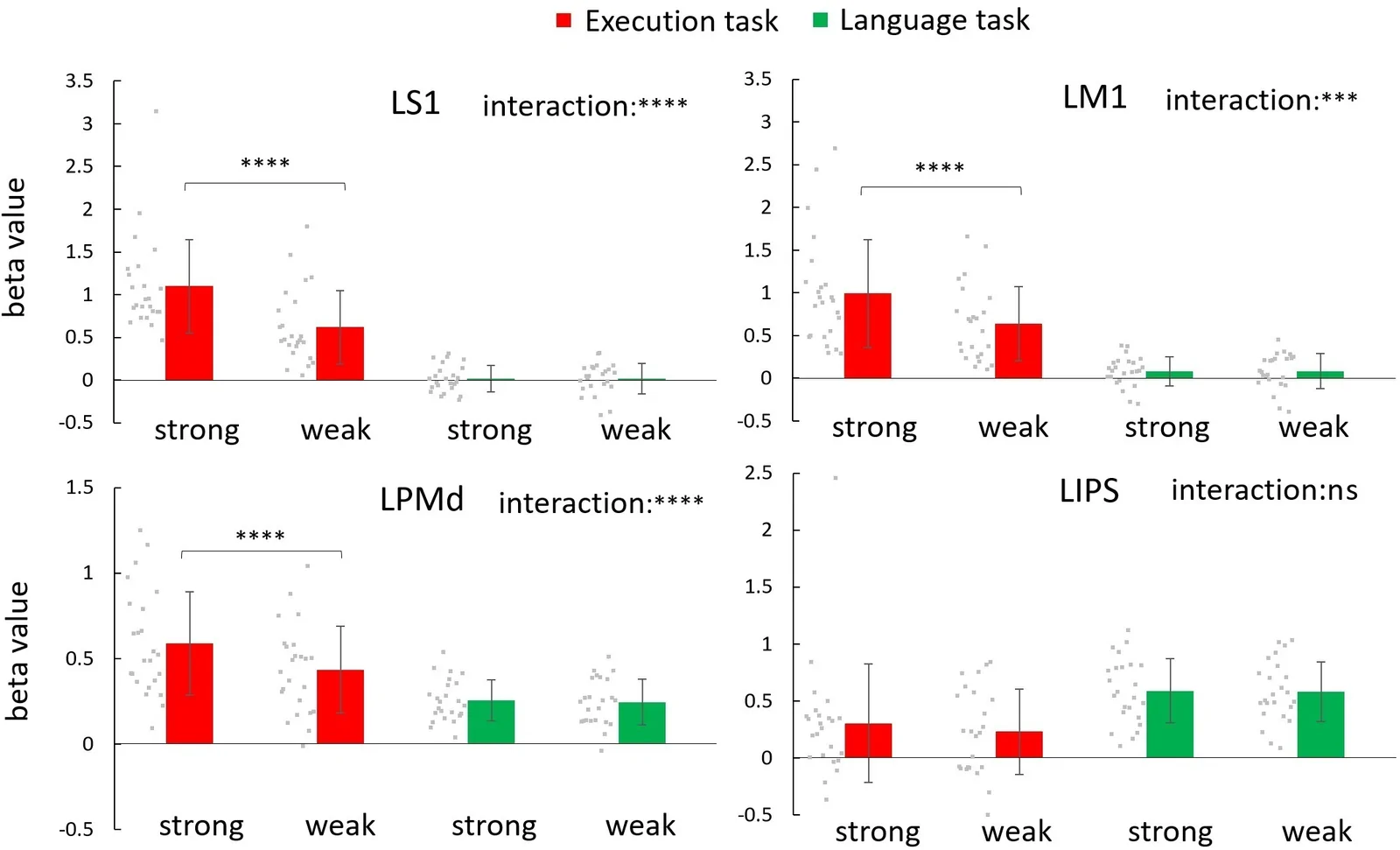
Mean beta value in each condition for each ROI. Gray dots show individual participant data. Error bars denote standard deviation. LS1, left primary somatosensory cortex; LM1, left primary motor cortex; LPMd, left dorsal premotor cortex; LIPS, left intraparietal sulcus; ns, not significant; ***p < 0.005; ****p < 0.001.

### 3.3 MVPA

#### 3.3.1 MVPA searchlight analysis

First, a whole-brain searchlight analysis was performed to examine the neural representations of action intensity. Within the execution task, the intensity classification analysis revealed a significant above-chance decoding accuracy across a widespread region centered in the left precentral and postcentral gyri extending to the supplementary motor area and distributed across the subcortical and cerebellar regions, including the bilateral thalamus, basal ganglia (the bilateral putamen, caudate nucleus, and globus pallidus), bilateral cerebellum, and bilateral hippocampus (Fig. 5; Supplementary Table S2). However, within the language task, a significant above-chance decoding accuracy was observed only for the LIPS (Fig. 6; Supplementary Table S2). For cross-task decoding, a cross-classification (direction-averaged) revealed no significant clusters. Supplementary analyses were also performed separately for each decoding direction. In the execution-to-language direction, a significant cluster was observed in the LMFG (Supplementary Fig. S1; Supplementary Table S2). No significant clusters were identified in the language-to-execution direction.

**Fig. 5.**
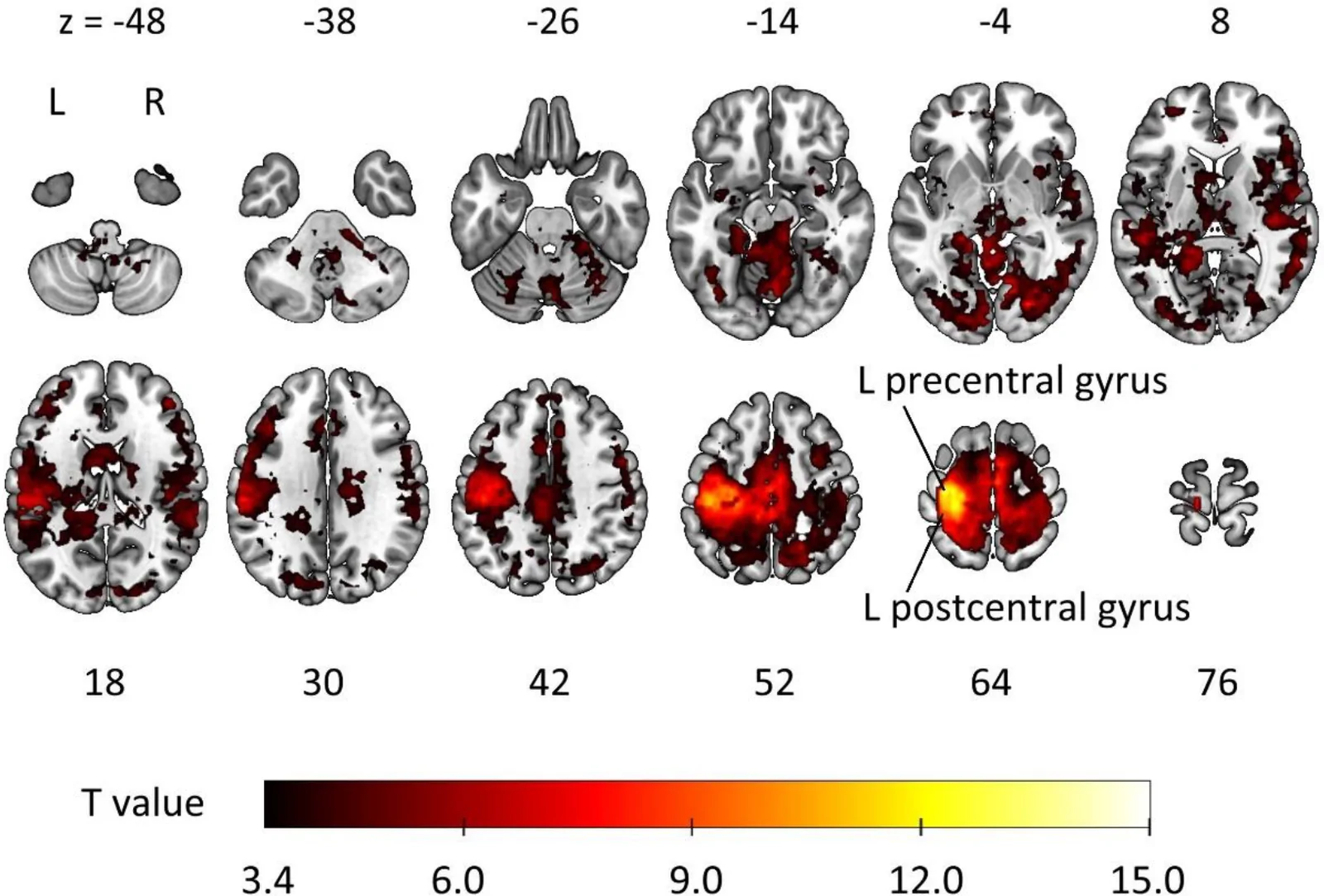
Brain regions showing distinct activation patterns between strong- versus weak-grip conditions in the execution task revealed by the searchlight analysis. Results were thresholded at *p* < 0.001 uncorrected at the voxel level and *p* < 0.05 FWE-corrected at the cluster level. L = Left; R = Right.

**Fig. 6.**
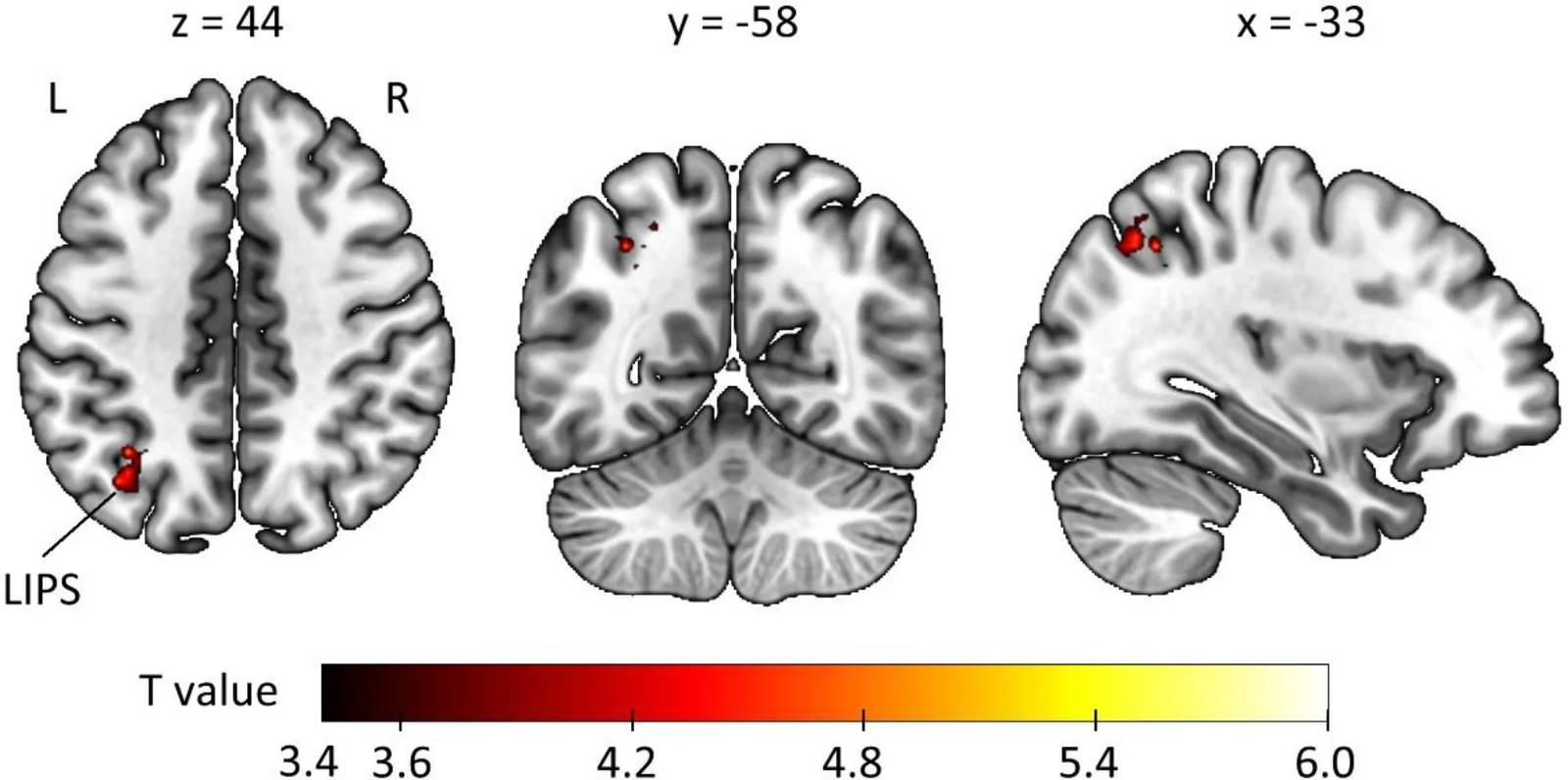
Brain region showing distinct activation patterns between strong- versus weak-grip conditions in the language task revealed by the searchlight analysis. Results were thresholded at *p* < 0.001 uncorrected at the voxel level and *p* < 0.05 FWE-corrected at the cluster level. L = Left; R = Right.

In addition, to assess the robustness of the searchlight results across spatial scales, an additional searchlight analysis using a 9-mm radius was conducted. Within the execution task, the results were broadly similar to those obtained with the 12-mm searchlight analysis (Supplementary Fig. S2). In contrast, no significant clusters were found in the language task or in any cross-classification analyses.

Finally, exploratory whole-brain searchlight maps were generated for the language decoding and cross-classification (direction-averaged) at a liberal threshold (*p* < 0.05, uncorrected for multiple comparisons). These maps were intended to illustrate the broader spatial distribution of decoding effects beyond the corrected threshold (Supplementary Figs. S3 and S4). The language decoding analysis revealed clusters in regions that included the posterior portions of the LIPS. In the cross-classification analysis, a more spatially restricted pattern was observed, with clusters emerging in a region including the posterior LIPS and showing a tendency toward more medial locations within this region, together with smaller clusters in additional regions.

#### 3.3.2 MVPA ROI analysis

Figure 7 shows the decoding results for the 12-mm ROIs in the LS1, LM1, LPMd, and LIPS for the execution, language, and direction-averaged cross-decoding analyses, as well as the corresponding results for the 10-mm and 15-mm ROIs used for robustness checks. The full set of decoding results across functional ROI sizes (12, 10, and 15 mm) and decoding conditions (execution, language, direction-averaged cross-decoding, execution-to-language cross-decoding, and language-to-execution cross-decoding) is summarized in Supplementary Tables S3–S5, including the classification accuracy, effect sizes, and Bayes factors.

**Fig. 7.**
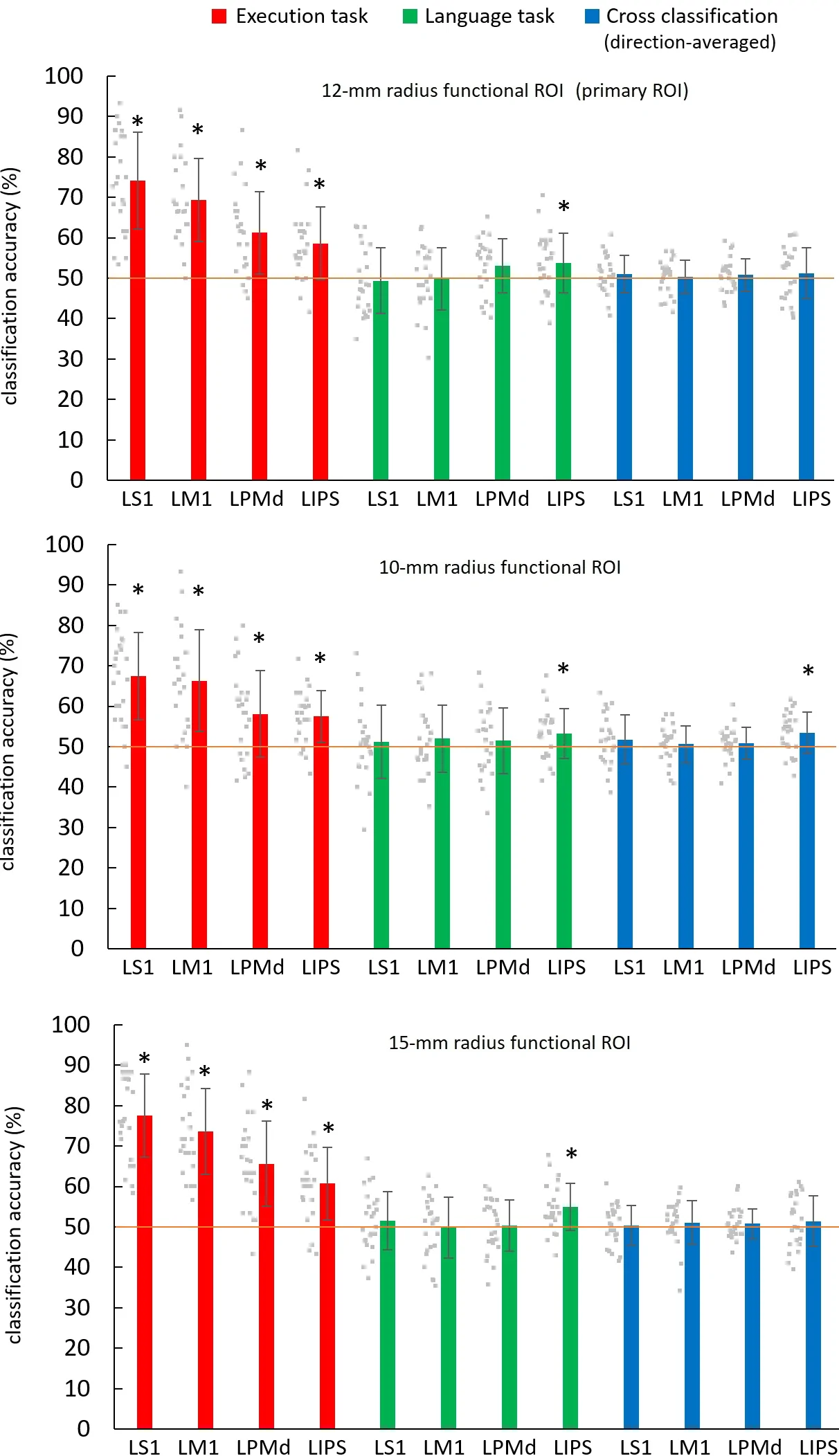
Mean classification accuracy in each condition for spherical ROIs of different radii (10, 12, and 15 mm). Gray dots show individual participant data. Error bars denote standard deviation. The orange line denotes the chance level of classification (50%). *p < 0.05, corrected for multiple comparisons across ROIs.

For the 12-mm ROIs, all ROIs in the execution task exhibited classification accuracy significantly above chance (chance level = 50%) with correction for multiple comparisons across ROIs, with large effect sizes and Bayes factors indicating strong evidence for above-chance decoding. In the language task, only the LIPS showed classification accuracy significantly above chance, with a moderate effect size (*d* = 0.50) and a Bayes factor indicating moderate evidence for above-chance decoding (*BF*_+0_ = 4.94). The LPMd showed above-chance decoding only at the uncorrected level, with a moderate effect size (*d* = 0.45) and a Bayes factor indicating some evidence for above-chance decoding (*BF*_+0_ = 3.19). For the cross-classification (direction-averaged), no significant effects were observed in any ROI, even without correction for multiple comparisons. The effect sizes were small and the Bayes factors were consistently below 1, providing evidence against reliable cross-task decoding. Similar patterns were observed for the directional cross-decoding analyses (execution-to-language and language-to-execution), with no significant effects, small effect sizes, and Bayes factors below 1 across all ROIs.

Additional ROI analyses were conducted using radii of 10 and 15 mm for the same four regions. For the 10-mm ROIs, all four regions showed significant above-chance accuracy in the execution task, whereas only the LIPS showed significant above-chance accuracy in the language task, which is consistent with the 12-mm ROI results. However, unlike the 12-mm ROI results, the cross-classification (direction-averaged) revealed above-chance accuracy in the LIPS, with a moderate effect size (*d* = 0.65) and a Bayes factor indicating strong evidence for above-chance decoding (*BF*_+0_ = 20.86). In the direction-specific analyses for the LIPS, the execution-to-language direction showed above-chance decoding at an uncorrected threshold, with a moderate effect size (*d* = 0.45) and a Bayes factor indicating some evidence for above-chance decoding (*BF*_+0_ = 3.03). For the language-to-execution direction, significant above-chance decoding was observed after correction for multiple comparisons, with a moderate effect size (*d* = 0.53) and a Bayes factor indicating moderate evidence for above-chance decoding (*BF*_+0_ = 6.22). In the other regions, the LPMd showed significant cross-classification for the execution-to-language direction after correction (*d* = 0.54, *BF*_+0_ = 7.14), whereas the language-to-execution direction showed no significant above-chance decoding, with smaller effect sizes and Bayes factors below 1, indicating no reliable evidence for above-chance decoding.

For the 15-mm ROIs, the results were broadly consistent with those obtained with the 12-mm ROIs: all four regions showed significant above-chance accuracy in the execution task, whereas only the LIPS showed significant above-chance accuracy in the language task. No significant cross-classification effects were observed in any region after correction for multiple comparisons. Above-chance decoding at an uncorrected threshold was observed in the LM1 for the language-to-execution direction (*d* = 0.37, *BF*_+0_ = 1.61), providing only anecdotal evidence for above-chance decoding.

#### 3.3.3 MVPA using anatomical IPS ROIs

Additional ROI MVPA was conducted using anatomically defined LIPS subregions (left hIP1–hIP8) (Supplementary Fig. S5; Supplementary Table S6). In the execution task, the left hIP1, hIP2, hIP5, hIP6, and hIP8 showed significant above-chance decoding after correction for multiple comparisons across the eight ROIs, whereas the left hIP3 and hIP7 showed above-chance decoding at an uncorrected threshold. All seven regions showed effect sizes exceeding 0.50 and Bayes factors greater than 6. In the language task, no regions survived correction for multiple comparisons; however, the left hIP3 and hIP6 showed above-chance decoding at an uncorrected threshold. These effects were accompanied by moderate effect sizes (hIP3: *d* = 0.50; hIP6: *d* = 0.43) and Bayes factors indicating moderate and anecdotal evidence, respectively (hIP3: *BF*_+0_ = 4.72; hIP6: *BF*_+0_ = 2.60). For cross-classification (direction-averaged), no regions showed significant decoding, including at an uncorrected threshold. The largest effect was observed in left hIP6 (*d* = 0.34, *BF*_+0_ = 1.29), whereas all other regions showed Bayes factors below 1, indicating evidence in favor of the null hypothesis. In the direction-specific analyses, left hIP6 showed above-chance decoding at an uncorrected threshold in the execution-to-language direction, with a moderate effect size (*d* = 0.37) and anecdotal evidence (*BF*_+0_ = 1.68).

### 3.4 Control analysis

#### 3.4.1 Control analyses for behavioral variability in grip force

A significant pre–post difference was observed in the 10% (weak-grip) condition in the behavioral results. All participants exhibited a higher grip force in the post-experiment weak condition than in the pre-experiment measurement (see Supplementary Fig. S6 for individual pre–post deviations). We examined whether this variability influenced the decoding performance. The ROI decoding analyses were repeated after excluding the 5 participants who showed the largest pre–post deviations in grip force, resulting in a reduced sample size of 19 participants. The means (*SD*s) of right-hand grip forces were as follows: pre-experiment 60% condition, 11.8 kg (3.0); pre-experiment 10% condition, 1.9 kg (0.5); post-experiment 60% condition, 10.5 kg (2.5); post-experiment 10% condition, 3.7 kg (1.4). A two-way ANOVA revealed a significant interaction between measurement phase and force intensity, *F*(1, 18) = 22.50, *p* < 0.001, η_p_² = 0.56, as well as a significant main effect for force intensity level *F*(1, 18) = 361.36, *p* < 0.001, η_p_² = 0.95. Post hoc comparisons indicated that the 60% condition showed no significant pre–post difference (*F*(1, 18) = 4.32, *p* = 0.052, η_p_² = 0.19), but the 10% condition showed a significant pre–post difference (*F*(1, 18) = 40.30, *p* < 0.001, η_p_² = 0.69), even after excluding the five participants with the largest deviations.

Accordingly, MVPA was conducted on a smaller sample (*n* = 19). Excluding these five participants produced a broadly similar pattern of ROI decoding results to those observed in the full sample, with significant effects in the LIPS during the language task across ROI sizes (12, 10, and 15 mm) and a significant cross-classification effect (direction-averaged) observed only for the 10-mm ROI after correction (Supplementary Tables S7–S9).

In addition, a correlation analysis (*n* = 24) was conducted to examine the relationship between the magnitude of the pre–post deviation in the 10% (weak-grip) condition and the LIPS (12 mm) decoding accuracy in the execution, language, and cross-classification (direction-averaged) analyses (Supplementary Fig. S6). The results showed that none of the correlations were significant, and the overall correlation coefficients were small.

#### 3.4.2 Control analyses for task-order effects

Participants were divided into two groups based on task order (execution-first vs. language-first), and decoding accuracies were compared between the groups. Two-sample *t*-tests were conducted for each ROI (full results are provided in Supplementary Tables S10–S12). No significant differences between the groups were observed after correction for multiple comparisons across ROIs. At an uncorrected threshold, a group difference was observed only in the LPMd for the 12-mm ROI in the cross-classification.

## 4 Discussion

In the execution task, our results demonstrated that the LM1, LS1, LPMd, and LIPS showed distinct activation patterns for strong and weak grip execution, broadly consistent with previous findings reported by Caccialupi et al. (2025); however, that study examined continuous relationships with force intensity using support vector regression, whereas the present study focused on binary classification between strong- and weak-grip conditions. In that study, the precise extent of the regions showing increased activation for strong gripping beyond LM1 and LS1 was not explicitly described, leaving the details unclear. In the present study, the activation level was significantly greater for strong grip across a broad area encompassing the LM1, LS1, and LPMd, whereas no such modulation by grip intensity was observed in the LIPS. In summary, the LM1, LS1, and LPMd all showed significantly greater activation for strong-grip than for weak-grip executions, and their multivoxel activity patterns successfully discriminated between the two grip intensities. In contrast, the LIPS exhibited distinct multivoxel patterns; however, the univariate activation level did not differ significantly between the two intensities. These findings suggest that action intensity is represented in the LM1, LS1, and LPMd in terms of univariate activation levels and multivoxel pattern differences, whereas in the LIPS, it may be represented primarily via pattern differences.

The PMd and IPS are recognized as regions involved in motor planning before the actual execution of movement in the context of motor execution (Ariani et al., 2015, 2025; Caccialupi et al., 2025; Gallivan et al., 2011a, 2011b, 2013, 2016; Pilacinski et al., 2018). Previously, Caccialupi et al. (2025) used MVPA and demonstrated that prior to executing right-hand actions of varying intensities, the LIPS and LPMd are involved in transforming visual cues regarding intensity differences into corresponding motor plans. They further argued that the IPS encodes motor plans and acts as a hub that converts abstract action goals into concrete motor programs. Meanwhile, the PMd maintains these motor plans, and the M1 contributes primarily to motor output rather than to the maintenance of predicted motor plans. Based on previous accounts, the present findings may reflect a functional organization in which the LIPS is involved in supporting execution-related representations in motor regions such as the LM1.

In the language task, no increase in univariate activation levels for the strong condition was observed in any of the four functional ROIs. In the MVPA, the LPMd showed above-chance decoding only at an uncorrected level in the primary 12-mm ROI analysis. In contrast, the LIPS showed reliable discrimination between strong and weak conditions across all ROI sizes, with significant effects observed after correction for multiple comparisons, suggesting relatively robust decoding of action intensity during language comprehension. The searchlight analysis also revealed significant clusters in regions proximal to the LIPS ROI. However, language-task decoding alone does not indicate whether these representations are shared with those involved in action execution. Therefore, cross-classification analyses (direction-averaged) were conducted. No significant cross-classification effects were observed in the primary 12-mm ROI, 15-mm ROI, or searchlight analysis. However, significant cross-classification was observed in the 10-mm ROI, which was included as a validation analysis, accompanied by a moderate effect size and strong Bayes-factor evidence.

Taken together, the present results indicate that the LIPS exhibited distinct activity patterns depending on the grip intensity in both the execution and language tasks. Furthermore, cross-classification effects (direction-averaged) were observed only in the 10-mm LIPS ROI. These findings suggest that the representations shared between execution and language, if present, may be spatially restricted.

The IPS extends along the anterior–posterior axis and is known to exhibit functional heterogeneity across its subregions. For example, previous studies have suggested that posterior parietal subregions differ in effector specificity (Leoné et al., 2014; Heed et al., 2016). Because the spherical functional ROI may not fully capture the extent of the IPS, additional analyses were conducted using anatomically defined ROIs (left hIP1–hIP8). Although no significant cross-classification effects were observed in these subregions, the left hIP6 showed somewhat larger effect sizes and Bayes factors than the other subregions. However, the center of the functional LIPS ROI used in the present study was located within hIP3 rather than hIP6. In addition, supplementary exploratory searchlight analyses using a liberal threshold (*p* < 0.05, uncorrected) revealed small clusters within the posterior IPS. Although no direct evidence was obtained regarding the precise localization of shared representations within the IPS, the present findings leave open the possibility that such representations may be spatially restricted within the posterior IPS. In line with previous work showing that decoding performance can vary depending on the ROI size (Coutanche et al., 2016), one possible explanation is that shared neural activity is concentrated within a relatively small subregion of the posterior LIPS, whereas larger ROIs may introduce additional noise and reduce classification performance.

The present findings are discussed from the perspective of embodied cognition. From the perspective of a strong version of this framework, neural activity similar to that observed during action execution would be expected during language-based action comprehension, particularly in lower-level sensorimotor regions, such as M1 and S1. Because the PMd and IPS are involved in a variety of cognitive processes beyond motor control, shared representations in these regions alone would provide only limited support for an embodied account.

In the present study, no increase in univariate activation levels for the strong condition was observed in the LM1 or LS1, and the cross-classification analysis (direction-averaged) did not reveal significant discrimination between the strong and weak conditions in these regions. Similarly, no such increase was observed in the LPMd and LIPS, which are relatively higher-order action-related regions. In terms of multivoxel patterns, the LPMd showed above-chance decoding during the language task only at an uncorrected level in the primary 12-mm ROI analysis, whereas no significant cross-classification effects (direction-averaged) were observed. In contrast, the LIPS showed robust decoding during the language task across ROI sizes and cross-classification effects in the 10-mm ROI, although the primary 12-mm ROI did not show significant cross-classification effects. These findings suggest that the LIPS may contain partially shared representations related to action intensity.

The PMd and IPS have been implicated in motor preparation and action planning (Ariani et al., 2015, 2025; Caccialupi et al., 2025; Gallivan et al., 2011a, 2011b, 2013, 2016; Pilacinski et al., 2018). In addition, the ROIs used in the present study were defined based on a previous study demonstrating the decoding of action execution intensity (Caccialupi et al., 2025). Therefore, it is possible that higher-order action-related representations are involved in both action execution and comprehension of the action intensity conveyed by sentences. Nevertheless, the present findings do not necessarily indicate that language comprehension directly reinstates the neural representations closely tied to motor execution.

In addition to action-related functions such as motor planning, the IPS is also known to support a variety of other functions, including numerical processing, visual working memory, and the representation of semantic similarity (Bulthé et al., 2014, 2015; Damarla & Just, 2013; Eger et al., 2009; Harvey et al., 2013; Lyons & Beilock, 2018; Lyons et al., 2015; Neyens et al., 2017; Nieder, 2005; Piazza et al., 2004, 2007; Todd & Marois, 2004; Wilkey et al., 2020). Accordingly, these non-action-specific processes may have contributed to the comprehension of the action intensity conveyed by the sentences. More specifically, with regard to numerical processing, several studies have suggested that the parietal cortex represents magnitude information in various domains, such as time, space, and number (Bueti & Walsh, 2009; Pinel et al., 2004; Walsh, 2003). One MVPA study demonstrated that numerical magnitude information can be represented in the parietal cortex across different stimulus formats, such as dot arrays and digits (Wilkey et al., 2020), whereas other studies have emphasized format-dependent representations (Bulthé et al., 2014, 2015; Lyons & Beilock, 2018; Lyons et al., 2015). The current results raise the possibility that representations of action intensity or force magnitude may be encoded in the LIPS, potentially alongside other magnitude-related representations that have been reported in this region. It should be noted that in the execution task, action intensity was instructed using explicit numerical values; that is, participants were asked to perform “strong” and “weak” gripping at 60% and 10% of their MVC, respectively. Thus, it remains possible that the present task implicitly engaged in numerical processing, particularly in the execution task, raising the possibility that the observed representations partly reflected number-related magnitude coding rather than purely action-specific intensity magnitude coding.

Previous studies reported that sentences describing actions requiring greater physical effort, such as moving a sofa rather than a picture, elicit greater activation in the LMFG, LIFG, and LIPL (Moody & Gennari, 2010; Urrutia et al., 2012). These manipulations varied in the degree of involvement across body parts, including the legs and torso. In contrast, the present study manipulated only the intensity of right-hand gripping actions through adverbial expressions (e.g., firmly, lightly), thereby isolating action intensity from broader whole-body movement contexts. Our results revealed that the neural representations underlying the comprehension of action intensity in sentences describing right-hand gripping actions were encoded in the activity patterns of the LIPS. Recent work has shown that effector-specific parametric information about grip force can be decoded from the contralateral IPS during the early stages of action preparation, whereas decodable representations in M1 emerge at a later stage closer to movement execution (Caccialupi et al., 2026). Therefore, it is possible that the LIPS activity observed in the present study reflects effector-dependent representations associated with right-hand actions. However, the present study did not include conditions involving the left hand, either in action execution or in language stimuli, and, therefore, does not allow us to determine whether the observed LIPS representations are truly effector-specific. Previous MVPA studies have demonstrated that parieto-frontal regions can encode both effector-dependent and effector-independent action information (Gallivan et al., 2011a). Accordingly, an alternative possibility is that the IPS encodes more abstract representations of intensity that are not tied to a specific effector. Distinguishing between effector-dependent and effector-independent representations in the IPS remains an important question for future research.

Taken together, the present findings suggest that partially shared representations between action execution and language comprehension may exist within a spatially restricted region of the LIPS. In addition, the observed LIPS activity might reflect representations related to the intensity of right-hand actions. However, alternative interpretations remain possible, including the view that the LIPS represents more abstract magnitude-related information extending beyond action-specific processing. Accordingly, although the present study cannot determine whether these representations are specific to action processing, the findings suggest that higher-level representations of intensity in the LIPS may be related to the comprehension of sentences describing action intensity and may also be partially shared across action execution and language comprehension. Overall, the present findings do not provide strong support for the view that the comprehension of sentences describing action intensity is grounded in the direct reinstatement of low-level motor execution processes, as predicted by strong versions of embodied cognition.

In addition to ROI-based analyses, whole-brain analyses revealed effects outside the pre-defined ROIs. Specifically, in the execution condition, greater activation of strong compared with weak grips was observed in the cerebellum and subcortical regions, including the thalamus, basal ganglia, and hippocampus. A searchlight MVPA revealed a similar pattern, showing that grip force could be decoded from these regions. Notably, these effects were restricted to the execution condition and were not observed in the language task or cross-classification analysis. Previous studies have reported increased activity in cortical and cerebellar regions during movements requiring higher force (Dai et al., 2001; Keisker et al., 2009). In addition, multivariate analyses have shown that grip force can be decoded from widespread cortical areas (Caccialupi et al., 2025, 2026), and their reported maps suggest that force-related decoding effects may also extend beyond the cortex, including the cerebellum. Although subcortical regions have been less consistently reported in previous studies, this may partly reflect methodological differences as prior studies have often focused on cortical regions of interest. The basal ganglia play a well-established role in controlling motor output (Turner & Desmurget, 2010). Activity in specific basal ganglia nuclei and thalamic regions has been shown to increase with greater force amplitude during motor execution (Spraker et al., 2007). The present findings are broadly in line with those of previous studies implicating subcortical and cerebellar regions in force-dependent motor processing during execution. In contrast, the hippocampal effects are less likely to primarily reflect sensorimotor processing per se. Participants in the present study learned the associations between visual cues and force levels prior to scanning. The hippocampus has been implicated in associative memory (Eichenbaum, 2017; Mattfeld & Stark, 2015). Therefore, the observed hippocampal activation may reflect learned associations between visual cues and force levels.

To further examine the cross-decoding results, supplementary analyses were conducted for the execution-to-language and language-to-execution directions at both the whole-brain searchlight and ROI levels. In the LIPS ROI analyses, the results were broadly consistent between the direction-averaged and direction-specific analyses. Specifically, in the 10-mm ROI, significant effects were observed for the execution-to-language direction at an uncorrected threshold, whereas the language-to-execution direction remained significant after correction. In contrast, no significant effects were observed in the 12-mm and 15-mm ROIs. Thus, the direction-specific analyses yielded a pattern similar to that observed in the direction-averaged analysis, providing converging evidence that any shared representations between action execution and language comprehension may be spatially restricted within a limited subregion of the LIPS.

Direction-specific effects were also observed. A whole-brain searchlight analysis revealed a cluster in the LMFG in the execution-to-language direction, whereas no significant effects were observed in the language-to-execution direction. In addition, functional ROI analyses revealed significant cross-decoding in the 10-mm LPMd ROI for the execution-to-language direction, with no corresponding effects in the language-to-execution direction or in the 12- or 15-mm ROIs. Cross-decoding asymmetries have been reported in previous studies and may arise even when conditions share common information, due to differences in signal-to-noise ratio or the presence of condition-specific information across tasks (van den Hurk & Op de Beeck, 2019). Separately, cross-classification between different task contexts may also be influenced by variability unrelated to shared representational content, even when decoding appears successful (Hebart & Baker, 2018). Thus, the present cross-decoding results may have been influenced by factors beyond shared representational structure, such as differences in signal-to-noise ratio or task-specific information. In the present study, the execution and language tasks differed substantially in the experimental context, which may have led to unstable or asymmetric cross-decoding results. The PMd is implicated in motor planning and preparatory processes, although it is also known to be involved in non-action-related processes, whereas the MFG has been associated with cognitive control and working memory (Miller & Cohen, 2001; Petrides, 2005). The observed effects may, therefore, reflect processes associated with motor planning in the LPMd and cognitive control in the LMFG, both of which may be engaged across the two tasks. However, given the absence of corresponding effects in the language-only, direction-averaged, and language-to-execution direction analyses, the functional interpretation of the effects observed in the LPMd (ROI analysis) and LMFG (whole-brain searchlight analysis) remains uncertain and should be treated with caution.

Exploratory whole-brain searchlight analyses at a threshold of *p* < 0.05 (uncorrected for multiple comparisons) revealed that both language decoding and cross-classification effects were observed in the posterior portions of the LIPS, with the cross-classification effects possibly showing a tendency toward more medial locations within this region. Because these findings were obtained using a liberal uncorrected threshold, they should be interpreted with caution. Further studies are needed to determine the precise spatial organization of shared representations within this region.

Behavioral results showed that grip force was significantly higher in the 60% condition than in the 10% condition in both the pre- and post-experiment measurements, indicating that the two force conditions were distinguishable across phases. However, while no significant difference was observed between the pre- and post-experiment measurements in the 60% condition, the 10% condition showed a significantly higher force after the experiment than before (pre: 1.9 kg; post: 4.2 kg). One possible explanation for this difference is that the measurement procedures differed between the pre- and post-experiment measurement. In the pre-experiment measurements, participants received visual feedback and adjusted their grip force while monitoring a visual scale. In contrast, in the post-experiment measurement, no visual feedback was provided, and participants performed the task without viewing the scale. Grip force was not measured or fed back to participants during the experimental task. Three possibilities may account for the observed shift: (1) grip force in the weak condition gradually drifted toward higher values during the task, (2) in the absence of feedback, grip force in the weak condition was already higher than the pre-experiment level from the beginning of the task, or (3) grip force in the weak condition increased specifically during the post-experiment measurement. However, the present study cannot distinguish between these possibilities. It is important to note that the 10% condition required very low force (mean pre-experiment value: 1.9 kg). Because the task and post-experiment measurements were conducted without visual feedback, participants had no visual cues to verify whether they were producing such a low level of force, which may have biased the responses toward higher force levels. Indeed, all participants exhibited an increase in grip force in the post-experiment measurements (see Supplementary Fig. S6 for individual pre–post differences).

The inability to fully control for this deviation in the weak condition represents a limitation of this study. This concern is particularly relevant if the deviation reflects a gradual drift in grip force during the task, as such drift could systematically influence the neural patterns. To examine the potential influence of this deviation on the MVPA results, an additional analysis was conducted in which the five participants with large pre–post deviations were excluded, and ROI-based MVPA was performed on the remaining 19 participants. Although the tendency for higher post-experiment force in the 10% condition remained even after excluding these participants, different results would be expected between the full sample (*n* = 24) and the reduced sample (*n* = 19) if the magnitude of deviation substantially affected MVPA performance. Overall, the results were largely consistent after excluding five participants. Specifically, the cross-decoding analysis (direction-averaged) showed a significant effect only in the LIPS for the 10 mm ROI, with a medium effect size and relatively strong Bayes-factor evidence. Furthermore, correlations between decoding accuracy in the 12 mm LIPS ROI and the magnitude of pre–post deviation were examined for the execution, language, and cross-decoding conditions (direction-averaged); however, none of the correlations were significant, and no consistent pattern was observed across conditions. While the cross-decoding condition showed a modest negative correlation, the execution and language conditions showed near-zero correlations, suggesting that the observed association is unlikely to reflect a systematic relationship. Thus, these additional analyses suggest that the deviation in grip force in the 10% condition is unlikely to have substantially influenced MVPA performance. However, because grip force was not directly measured during task performance, it is not possible to strictly verify the correspondence between actual grip force and neural activity during the task. This remains a limitation of the present study.

Finally, to examine the possibility of carry-over or memory-related effects, participants were divided into two groups based on task order (language-first vs. execution-first), and the decoding accuracies were compared between the groups for each ROI. No significant differences between the groups were observed after correction for multiple comparisons. Although one ROI reached significance at the uncorrected level, this effect did not survive correction. Overall, these findings suggest that task order had little systematic influence on decoding performance and provided no clear evidence of carry-over or memory-related influences between tasks. However, both tasks were explained prior to scanning, and participants completed practice trials for both tasks before entering the scanner. Therefore, even in the absence of detectable order effects, it remains possible that prior exposure to one task influenced neural representations during the other task. For example, prior experience with the execution task may have strengthened motor simulation processes during the language task, whereas prior experience with the language task may have facilitated covert verbalization during the execution task. Future studies should more strictly dissociate task-specific representations by minimizing cross-task exposure prior to scanning.

## 5 Conclusion

In summary, cross-classification analyses revealed no evidence for shared neural representations between execution and language tasks in the primary (12 mm radius) ROI. However, ROI analyses using a smaller (10 mm radius) LIPS ROI showed significant cross-decoding performance with a moderate effect size. From the perspective of embodied cognition, the present findings do not support strong accounts assuming direct reinstatement of low-level motor execution processes during language comprehension. Instead, the results suggest that the comprehension of action intensity may be associated with higher-level intensity-related representations in the LIPS, which may be partially shared across action execution and language processing. Given the limitations of stimulus control and task design in the present study, further research is needed to clarify the nature of intensity representations in the IPS and the extent to which such representations are shared across language comprehension and action processing.

## Supplementary Material

Supplementary Material

## Data Availability

Participants consented to the disclosure of statistically processed data only. For this reason, raw data cannot be publicly shared. Unthresholded group-level statistical maps have been deposited in NeuroVault: https://identifiers.org/neurovault.collection:22421. The fMRI data were analyzed using standard procedures implemented in SPM. Multivariate pattern analysis was conducted using a linear support vector machine implemented in LIBSVM. Scripts used to organize the data and run the analyses were written in MATLAB and are available from the corresponding author upon reasonable request.

## Utilization of Generative AI and AI-Assisted Technologies in the Writing Process

The manuscript was drafted and refined using ChatGPT (OpenAI) to improve language editing and readability. The authors have reviewed and verified the scientific content and conclusions.
