# Supplementary material for: Role of the left intraparietal sulcus in the comprehension of action intensity described in sentences
Source: Imaging Neurosci (Camb). 2026 Sep 25;4:IMAG.a.1371. doi: 10.1162/IMAG.a.1371 (PMC13618355; doi:10.1162/IMAG.a.1371)
Supplement: Supplementary file 1 [file IMAG.a.1371_supp.pdf]

**Supplementary Material**

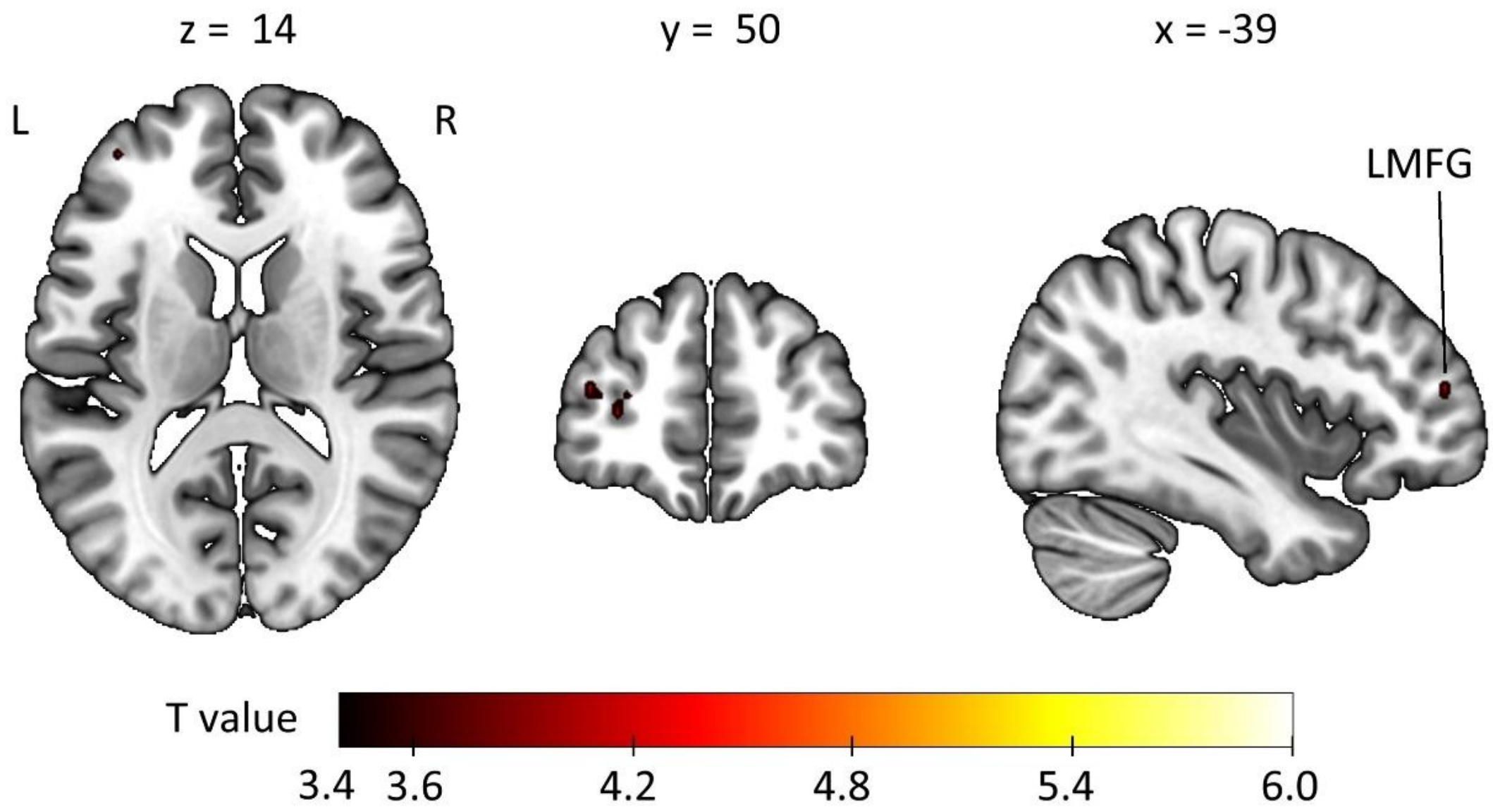


Supplementary Fig. S1. Brain region showing distinct activation patterns for cross-classification (from the execution task to the language task)

Results were thresholded at $p < 0.001$ uncorrected at the voxel level and $p < 0.05$ FWE-corrected at the cluster level. L = Left; R = Right.

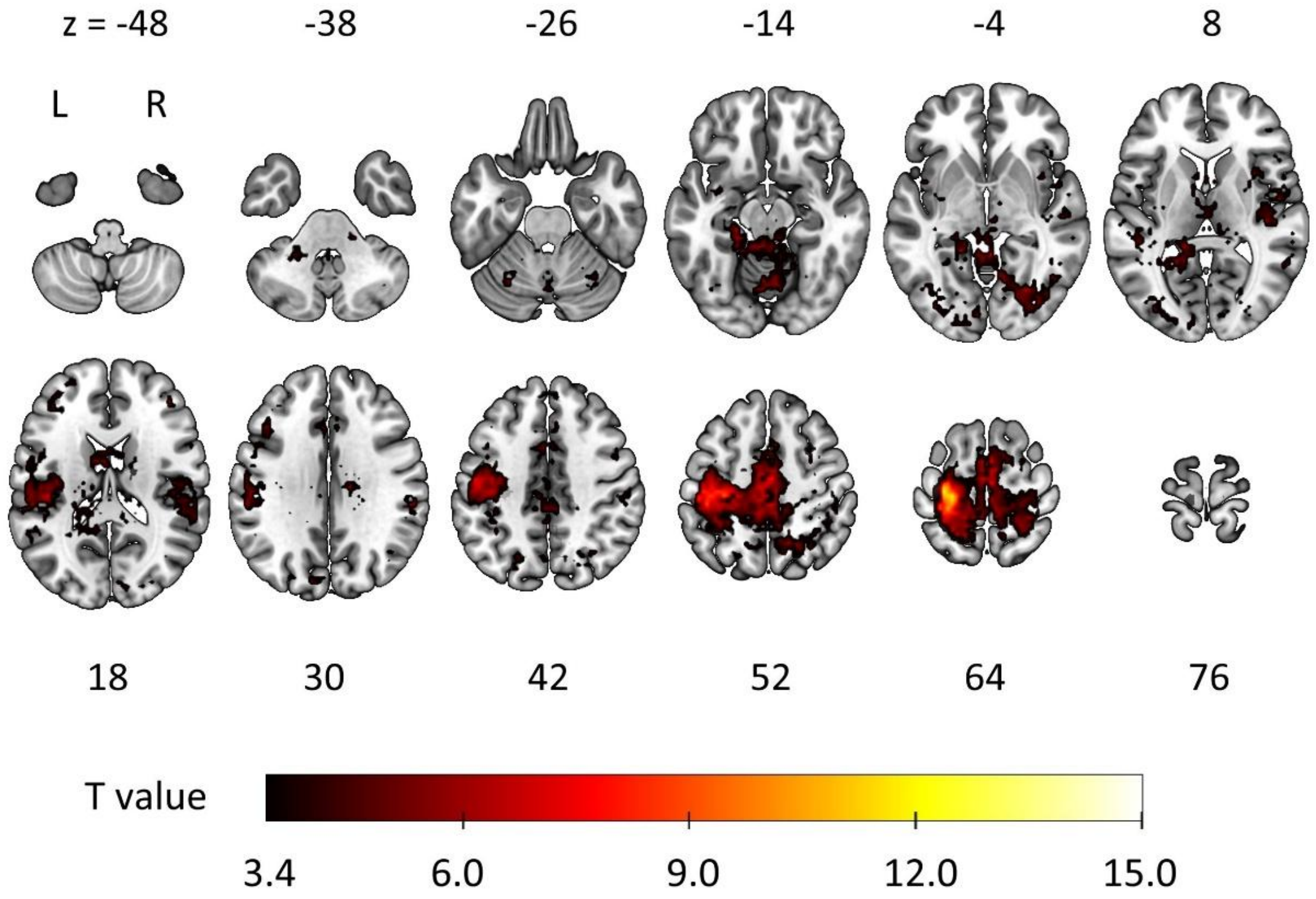


Supplementary Fig. S2. Brain regions showing distinct activation patterns between strong- versus weak-grip conditions in the execution task revealed by the supplementary searchlight analysis (9-mm radius)

Results were thresholded at $p < 0.001$ uncorrected at the voxel level and $p < 0.05$ FWE-corrected at the cluster level. L = Left; R = Right.

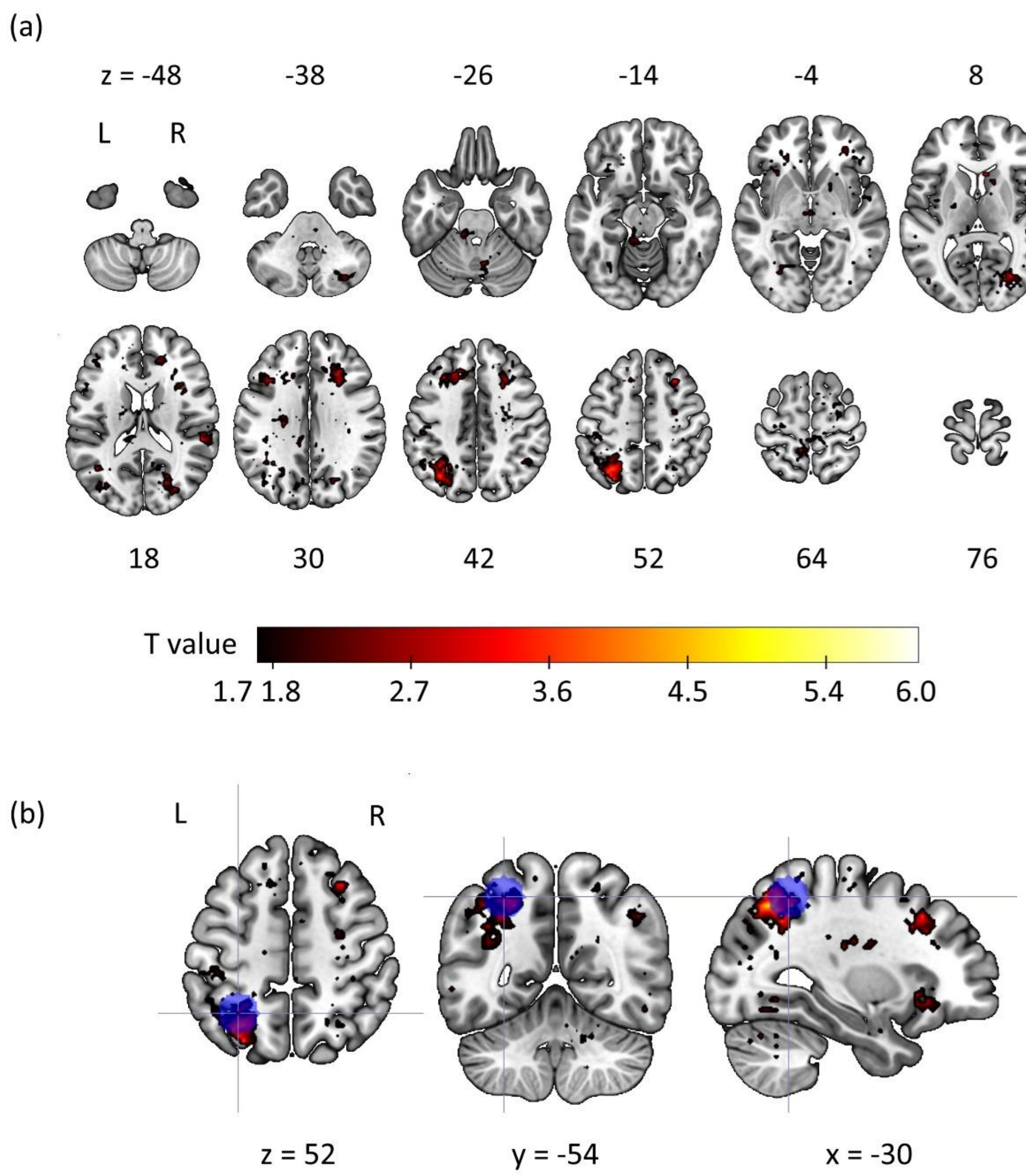


Supplementary Fig. S3. Whole-brain searchlight results for the language task (strong vs. weak grip conditions) at a liberal threshold

(a) Statistical maps are shown at $p < 0.05$ (uncorrected for multiple comparisons), without a minimum cluster size threshold. L = Left; R = Right.

(b) Three orthogonal slices showing the overlay of the functional IPS ROI (12-mm-radius sphere) and the statistical maps in (a) ($p < 0.05$, uncorrected, no minimum cluster size threshold).

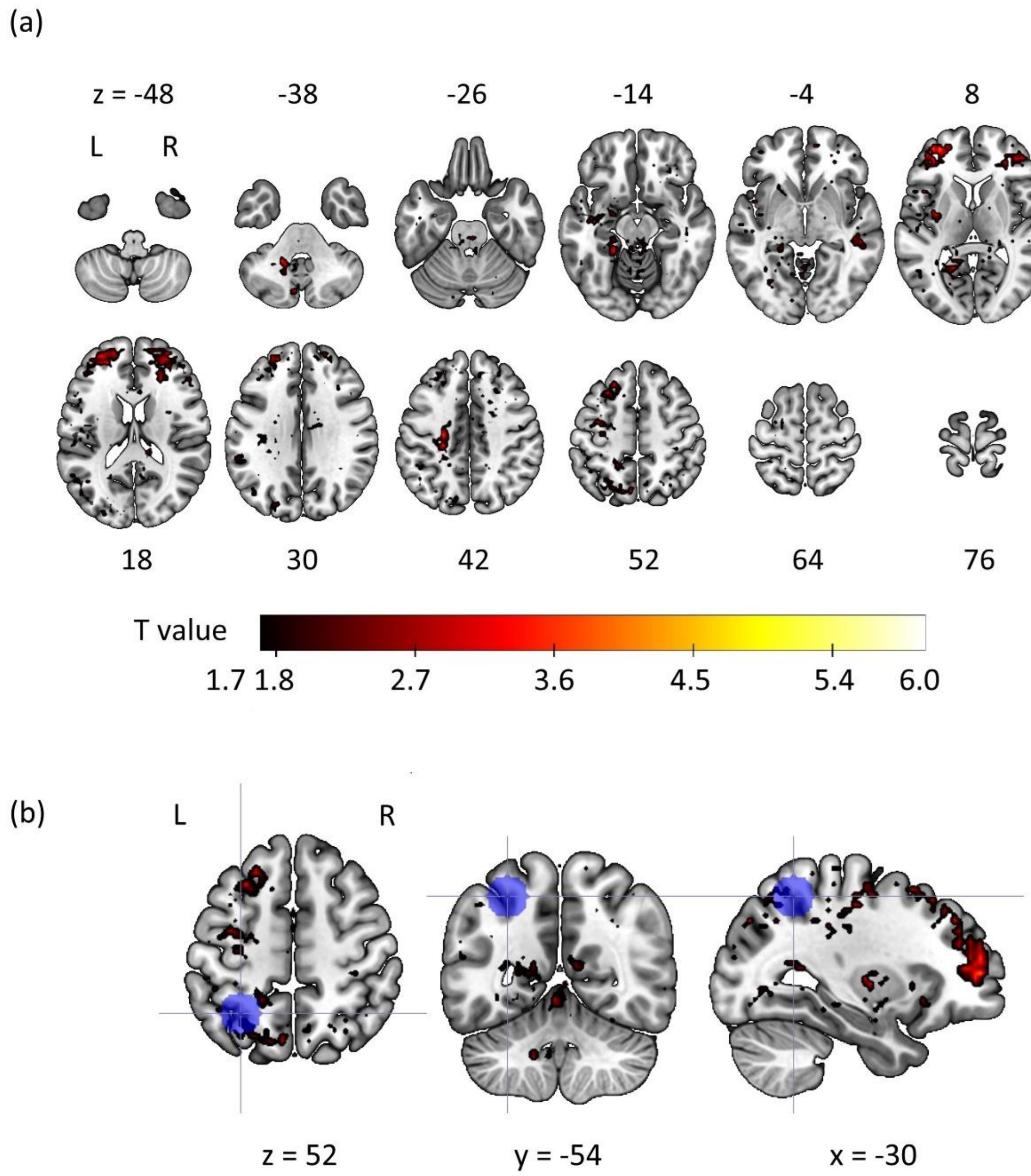


Supplementary Fig. S4. Whole-brain searchlight results for the cross classification (direction averaged) at a liberal threshold

(a) Statistical maps are shown at $p < 0.05$ (uncorrected for multiple comparisons), without a minimum cluster size threshold. L = Left; R = Right.

(b) Three orthogonal slices showing the overlay of the functional IPS ROI (12-mm-radius sphere) and the statistical maps in (a) ($p < 0.05$, uncorrected, no minimum cluster size threshold).

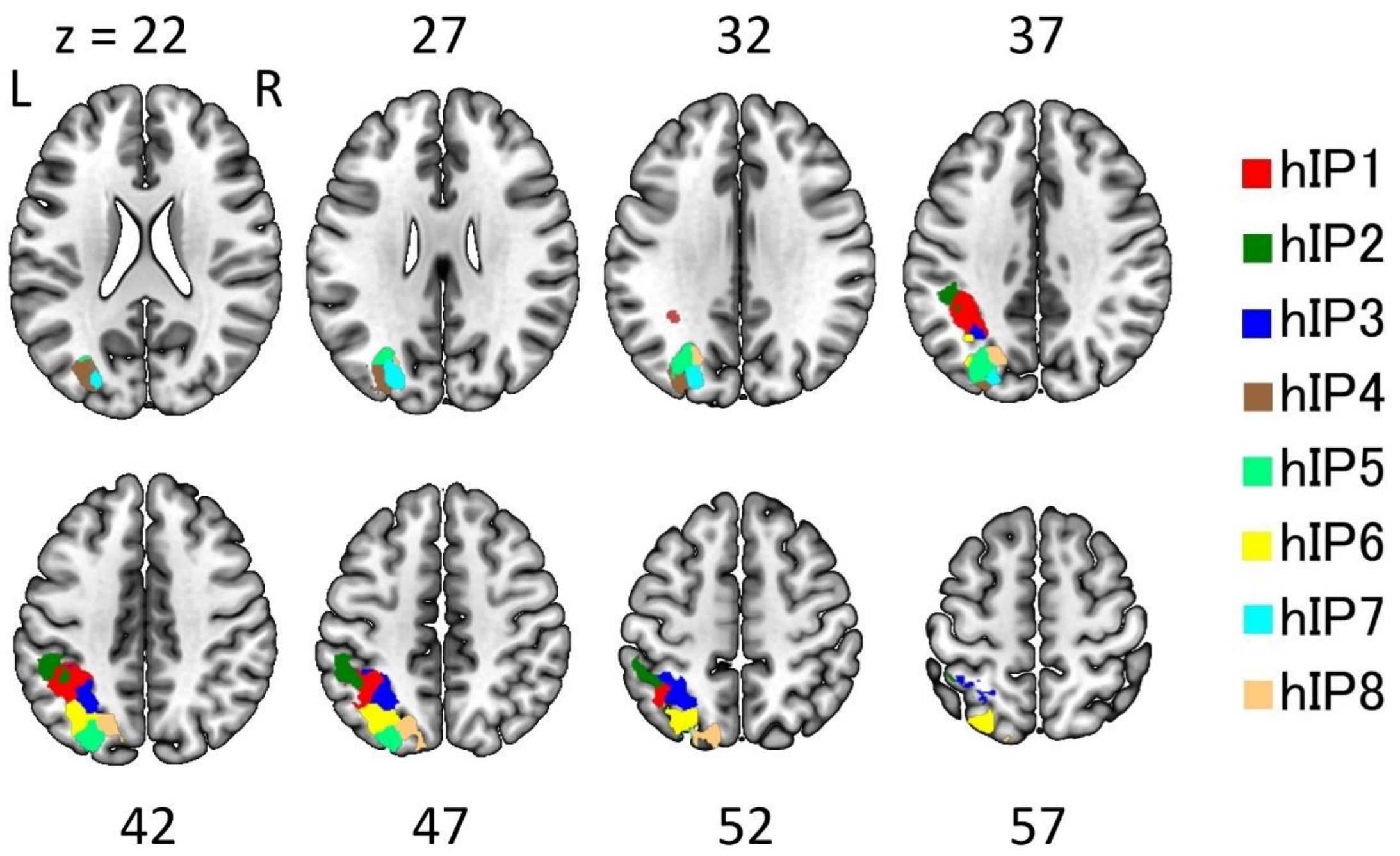


Supplementary Fig. S5. Intraparietal sulcus (IPS) subregions

Probabilistic cytoarchitectonic maps derived from the Anatomy Toolbox (version 3.0), where hIP1–hIP8 (human intraparietal areas 1–8) denote subdivisions along the IPS in the left hemisphere. L = Left; R = Right.

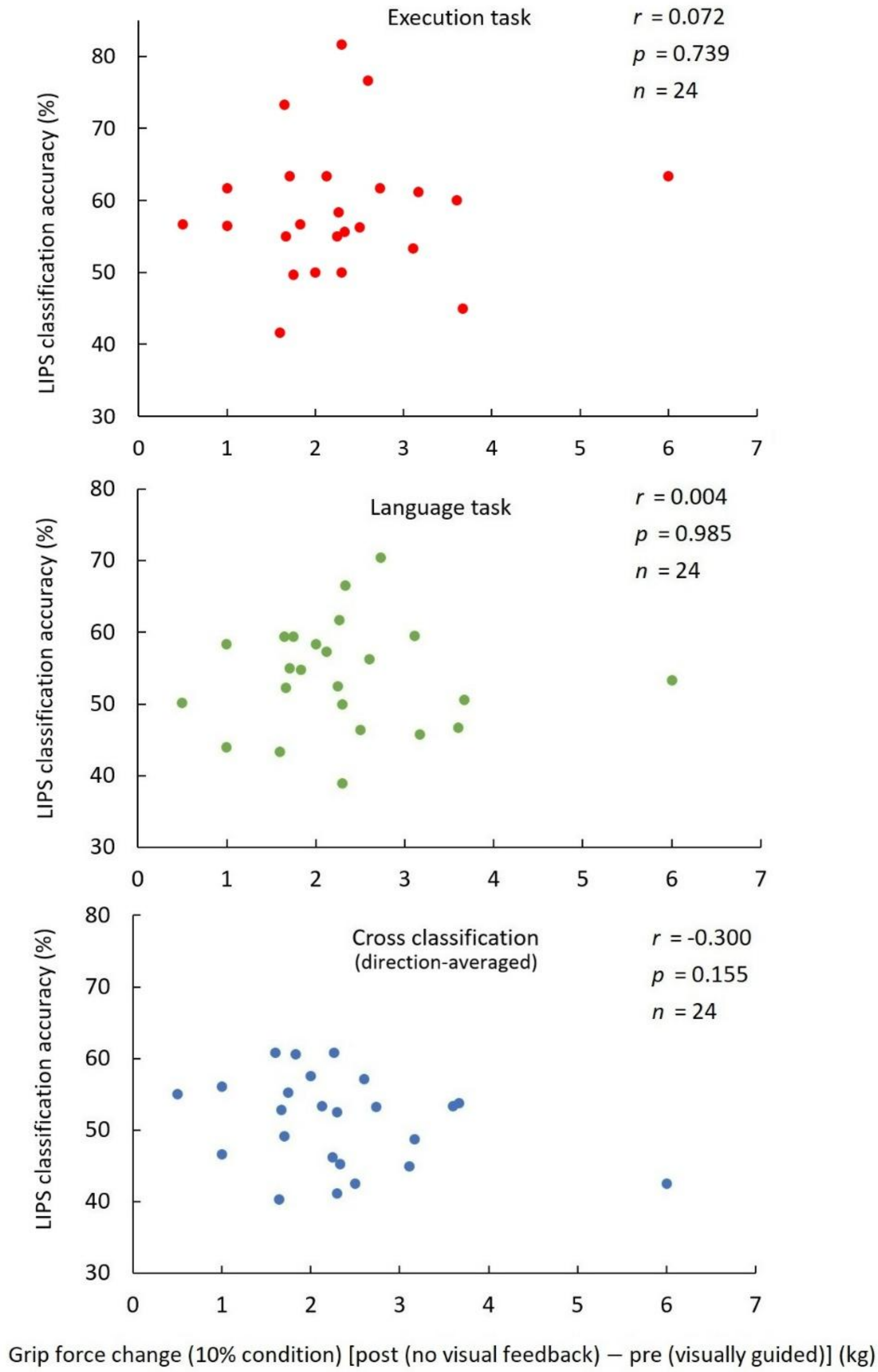


Supplementary Fig. S6. Relationships between pre–post change in grip force in the 10% (weak-grip) condition and decoding accuracy in the left intraparietal sulcus (LIPS) ROI Scatter plots illustrating the associations between the change in grip force in the weak condition (post: post-experiment measurement without visual feedback; pre: pre-experiment measurement with visual guidance) and decoding accuracies for the execution task, language task, and cross-classification (direction-averaged) within the subject-specific functional LIPS ROI (12 mm radius). Each dot represents one participant.

Supplementary Table S1. Peak MNI coordinates of brain regions revealed by univariate activity analysis

| Anatomic region | BA | MNI Coordinates x | y | z | Voxels | *t* value |
|---|---|---|---|---|---|---|
| Execution task: Strong-grip condition > Weak-grip condition | | | | | | |
| Left precentral gyrus | 6 | -33 | -25 | 62 | 4063 | 12.30 |
| Right Rolandic Operculum | - | 45 | -4 | 11 | 114 | 5.44 |

Results were thresholded at $p < 0.001$ uncorrected at the voxel level and $p < 0.05$ FWE-corrected at the cluster level.

BA, Brodmann area; MNI, Montreal Neurological Institute

Supplementary Table S2. Peak MNI coordinates of brain regions showing distinct activation patterns, as revealed by searchlight analysis

| Anatomic region | BA | MNI Coordinates | | | Voxels | *t* value |
|---|---|---|---|---|---|---|
| | | x | y | z | | |
| Execution task | | | | | | |
| Left precentral gyrus | 6 | -33 | -19 | 65 | 15878 | 14.39 |
| Right middle frontal gyrus | - | 21 | 47 | 32 | 25 | 5.34 |
| Language task | | | | | | |
| Left intraparietal sulcus | 7 | -33 | -58 | 44 | 44 | 5.24 |
| Cross-classification (Execution → Language) | | | | | | |
| Left middle frontal gyrus | 46 | -39 | 50 | 14 | 11 | 4.54 |

Results were thresholded at $p < 0.001$ uncorrected at the voxel level and $p < 0.05$ FWE-corrected at the cluster level.

Supplementary Table S3. MVPA results for 12-mm functional spherical ROIs ($n$ = 24) (primary ROI)

| ROI | Mean accuracy ± SD | t value | p value (one-tailed) | Effect size | $BF_{+0}$ |
|---|---|---|---|---|---|
| Execution task | | | | | |
| LS1 | 74.1 ± 12.0 | 9.65 | < 0.001* | 1.97 | $1.51 \times 10^{7}$ |
| LM1 | 69.3 ± 10.3 | 9.00 | < 0.001* | 1.84 | $4.62 \times 10^{6}$ |
| LPMd | 61.2 ± 10.2 | 5.29 | < 0.001* | 1.08 | 2067 |
| LIPS | 58.6 ± 9.0 | 4.56 | < 0.001* | 0.93 | 405 |
| Language task | | | | | |
| LS1 | 49.4 ± 8.1 | -0.38 | 0.646 | -0.08 | 0.17 |
| LM1 | 49.8 ± 7.7 | -0.11 | 0.542 | -0.02 | 0.20 |
| LPMd | 53.1 ± 6.7 | 2.21 | 0.019† | 0.45 | 3.19 |
| LIPS | 53.8 ± 7.4 | 2.45 | 0.011* | 0.50 | 4.94 |
| Cross-classification (direction-averaged) | | | | | |
| LS1 | 51.0 ± 4.6 | 1.06 | 0.150 | 0.22 | 0.60 |
| LM1 | 50.3 ± 4.1 | 0.31 | 0.379 | 0.06 | 0.28 |
| LPMd | 50.8 ± 4.0 | 0.93 | 0.180 | 0.19 | 0.51 |
| LIPS | 51.3 ± 6.2 | 0.97 | 0.171 | 0.20 | 0.54 |
| Cross-classification (Execution → Language) | | | | | |
| LS1 | 49.5 ± 3.7 | -0.70 | 0.753 | -0.14 | 0.14 |
| LM1 | 49.4 ± 4.9 | -0.60 | 0.723 | -0.12 | 0.14 |
| LPMd | 50.6 ± 3.8 | 0.82 | 0.210 | 0.17 | 0.45 |
| LIPS | 50.5 ± 6.4 | 0.37 | 0.359 | 0.08 | 0.29 |
| Cross-classification (Language → Execution) | | | | | |
| LS1 | 52.0 ± 7.1 | 1.37 | 0.092 | 0.28 | 0.88 |
| LM1 | 51.2 ± 5.9 | 0.94 | 0.178 | 0.19 | 0.52 |
| LPMd | 50.8 ± 6.0 | 0.62 | 0.269 | 0.13 | 0.37 |
| LIPS | 51.5 ± 8.7 | 0.82 | 0.210 | 0.17 | 0.45 |

*: $p < 0.05$ (one-tailed), corrected for multiple comparisons across the four ROIs; †: $p < 0.05$ (one-tailed), uncorrected.

Supplementary Table S4. MVPA results for 10-mm functional spherical ROIs (*n* = 24)

| ROI | Mean accuracy ± SD | t value | p value (one-tailed) | Effect size | $BF_{+0}$ |
|---|---|---|---|---|---|
| Execution task | | | | | |
| LS1 | 67.5 ± 10.8 | 7.77 | < 0.001* | 1.59 | 420100 |
| LM1 | 66.3 ± 12.6 | 6.21 | < 0.001* | 1.27 | 15780 |
| LPMd | 58.1 ± 10.6 | 3.66 | < 0.001* | 0.75 | 55.98 |
| LIPS | 57.4 ± 6.5 | 5.52 | < 0.001* | 1.13 | 3434 |
| Language task | | | | | |
| LS1 | 51.2 ± 9.1 | 0.62 | 0.272 | 0.13 | 0.37 |
| LM1 | 52.0 ± 8.3 | 1.14 | 0.134 | 0.23 | 0.65 |
| LPMd | 51.5 ± 8.1 | 0.87 | 0.197 | 0.18 | 0.48 |
| LIPS | 53.2 ± 6.1 | 2.51 | 0.010* | 0.51 | 5.43 |
| Cross-classification (direction-averaged) | | | | | |
| LS1 | 51.8 ± 6.1 | 1.39 | 0.090 | 0.28 | 0.90 |
| LM1 | 50.6 ± 4.5 | 0.65 | 0.262 | 0.13 | 0.38 |
| LPMd | 50.8 ± 4.0 | 0.99 | 0.166 | 0.20 | 0.55 |
| LIPS | 53.4 ± 5.1 | 3.19 | 0.002* | 0.65 | 20.86 |
| Cross-classification (Execution → Language) | | | | | |
| LS1 | 50.7 ± 4.7 | 0.71 | 0.244 | 0.14 | 0.40 |
| LM1 | 49.6 ± 4.4 | -0.46 | 0.677 | -0.10 | 0.16 |
| LPMd | 52.2 ± 3.9 | 2.65 | 0.007* | 0.54 | 7.14 |
| LIPS | 52.4 ± 5.4 | 2.18 | 0.020† | 0.45 | 3.03 |
| Cross-classification (Language → Execution) | | | | | |
| LS1 | 52.3 ± 8.7 | 1.24 | 0.113 | 0.25 | 0.75 |
| LM1 | 52.2 ± 6.8 | 1.55 | 0.067 | 0.32 | 1.14 |
| LPMd | 50.3 ± 6.5 | 0.20 | 0.421 | 0.04 | 0.25 |
| LIPS | 54.1 ± 7.6 | 2.58 | 0.008* | 0.53 | 6.22 |

*: $p < 0.05$ (one-tailed), corrected for multiple comparisons across the four ROIs; †: $p < 0.05$ (one-tailed), uncorrected.

Supplementary Table S5. MVPA results for 15-mm functional spherical ROIs (*n* = 24)

| ROI | Mean accuracy ± SD | t value | p value (one-tailed) | Effect size | $BF_{+0}$ |
|---|---|---|---|---|---|
| Execution task | | | | | |
| LS1 | 77.5 ± 10.3 | 12.84 | < 0.001* | 2.62 | $2.92\times10^{9}$ |
| LM1 | 73.6 ± 10.6 | 10.72 | < 0.001* | 2.19 | $9.93\times10^{7}$ |
| LPMd | 65.6 ± 10.6 | 7.08 | < 0.001* | 1.44 | 99650 |
| LIPS | 60.7 ± 8.9 | 5.74 | < 0.001* | 1.17 | 5551 |
| Language task | | | | | |
| LS1 | 51.5 ± 7.2 | 1.01 | 0.162 | 0.21 | 0.56 |
| LM1 | 49.8 ± 7.5 | -0.11 | 0.543 | -0.02 | 0.20 |
| LPMd | 50.3 ± 6.3 | 0.24 | 0.405 | 0.05 | 0.26 |
| LIPS | 54.9 ± 5.9 | 4.01 | < 0.001* | 0.82 | 120 |
| Cross-classification (direction-averaged) | | | | | |
| LS1 | 50.4 ± 4.8 | 0.38 | 0.355 | 0.08 | 0.29 |
| LM1 | 51.1 ± 5.3 | 0.98 | 0.168 | 0.20 | 0.54 |
| LPMd | 50.8 ± 3.7 | 1.04 | 0.155 | 0.21 | 0.58 |
| LIPS | 51.4 ± 6.3 | 1.07 | 0.149 | 0.22 | 0.60 |
| Cross-classification (Execution → Language) | | | | | |
| LS1 | 50.2 ± 4.1 | 0.25 | 0.404 | 0.05 | 0.26 |
| LM1 | 49.1 ± 4.2 | -0.96 | 0.827 | -0.20 | 0.12 |
| LPMd | 50.7 ± 3.3 | 1.00 | 0.164 | 0.20 | 0.56 |
| LIPS | 52.0 ± 6.9 | 1.39 | 0.089 | 0.28 | 0.91 |
| Cross-classification (Language → Execution) | | | | | |
| LS1 | 50.6 ± 7.0 | 0.43 | 0.335 | 0.09 | 0.31 |
| LM1 | 52.8 ± 7.6 | 1.79 | 0.043† | 0.37 | 1.61 |
| LPMd | 51.7 ± 6.2 | 1.32 | 0.100 | 0.27 | 0.83 |
| LIPS | 51.9 ± 7.0 | 1.32 | 0.100 | 0.27 | 0.83 |

*: $p < 0.05$ (one-tailed), corrected for multiple comparisons across the four ROIs; †: $p < 0.05$ (one-tailed), uncorrected.

Supplementary Table S6. MVPA results for anatomical IPS ROIs (*n* = 24)

| ROI | Mean accuracy ± SD | t value | p value (one-tailed) | Effect size | $BF_{+0}$ |
|---|---|---|---|---|---|
| Execution task | | | | | |
| hIP1 | 56.0 ± 9.5 | 3.02 | 0.003* | 0.62 | 14.54 |
| hIP2 | 55.2 ± 8.6 | 2.93 | 0.004* | 0.60 | 12.15 |
| hIP3 | 54.7 ± 8.7 | 2.58 | 0.008† | 0.53 | 6.21 |
| hIP4 | 52.3 ± 7.8 | 1.41 | 0.086 | 0.29 | 0.93 |
| hIP5 | 55.7 ± 6.9 | 3.96 | < .001* | 0.81 | 107 |
| hIP6 | 55.3 ± 8.8 | 2.91 | 0.004* | 0.59 | 11.78 |
| hIP7 | 54.4 ± 8.3 | 2.58 | 0.008† | 0.53 | 6.27 |
| hIP8 | 54.5 ± 7.3 | 2.93 | 0.004* | 0.60 | 12.20 |
| Language task | | | | | |
| hIP1 | 49.8 ± 5.3 | -0.14 | 0.554 | -0.03 | 0.19 |
| hIP2 | 51.9 ± 8.4 | 1.10 | 0.141 | 0.23 | 0.63 |
| hIP3 | 52.7 ± 5.4 | 2.43 | 0.012† | 0.50 | 4.72 |
| hIP4 | 50.0 ± 9.6 | 0.02 | 0.493 | 0.00 | 0.22 |
| hIP5 | 52.0 ± 8.1 | 1.16 | 0.13 | 0.24 | 0.67 |
| hIP6 | 53.2 ± 7.4 | 2.09 | 0.024† | 0.43 | 2.60 |
| hIP7 | 52.1 ± 8.2 | 1.23 | 0.116 | 0.25 | 0.73 |
| hIP8 | 49.0 ± 7.9 | -0.60 | 0.724 | -0.12 | 0.14 |
| Cross-classification (direction-averaged) | | | | | |
| hIP1 | 50.9 ± 4.1 | 1.08 | 0.145 | 0.22 | 0.61 |
| hIP2 | 50.5 ± 5.2 | 0.47 | 0.322 | 0.10 | 0.32 |
| hIP3 | 50.1 ± 5.6 | 0.07 | 0.472 | 0.02 | 0.23 |
| hIP4 | 50.0 ± 4.2 | 0.01 | 0.495 | 0.00 | 0.22 |
| hIP5 | 50.9 ± 5.8 | 0.78 | 0.221 | 0.16 | 0.44 |
| hIP6 | 51.5 ± 4.3 | 1.64 | 0.057 | 0.34 | 1.29 |
| hIP7 | 49.0 ± 4.1 | -1.24 | 0.886 | -0.25 | 0.11 |
| hIP8 | 49.3 ± 5.1 | -0.64 | 0.737 | -0.13 | 0.14 |
| Cross-classification (Execution → Language) | | | | | |
| hIP1 | 50.3 ± 5.6 | 0.23 | 0.41 | 0.05 | 0.26 |
| hIP2 | 50.2 ± 5.1 | 0.17 | 0.435 | 0.03 | 0.24 |
| hIP3 | 50.4 ± 5.8 | 0.35 | 0.366 | 0.07 | 0.29 |
| hIP4 | 49.1 ± 6.8 | -0.67 | 0.744 | -0.14 | 0.14 |
| hIP5 | 50.5 ± 6.4 | 0.34 | 0.367 | 0.07 | 0.28 |
| hIP6 | 52.2 ± 5.9 | 1.81 | 0.041† | 0.37 | 1.68 |
| hIP7 | 48.7 ± 4.6 | -1.38 | 0.909 | -0.28 | 0.10 |
| hIP8 | 49.4 ± 4.8 | -0.57 | 0.711 | -0.12 | 0.15 |
| Cross-classification (Language → Execution) | | | | | |
| hIP1 | 51.5 ± 6.0 | 1.21 | 0.119 | 0.25 | 0.72 |
| hIP2 | 50.7 ± 7.1 | 0.45 | 0.33 | 0.09 | 0.31 |

| | | | | | |
|---|---|---|---|---|---|
| hIP3 | 50.1 ± 7.9 | 0.08 | 0.47 | 0.02 | 0.23 |
| hIP4 | 50.7 ± 4.1 | 0.87 | 0.198 | 0.18 | 0.48 |
| hIP5 | 51.6 ± 5.7 | 1.33 | 0.099 | 0.27 | 0.83 |
| hIP6 | 50.3 ± 5.3 | 0.23 | 0.411 | 0.05 | 0.26 |
| hIP7 | 49.5 ± 7.5 | -0.31 | 0.619 | -0.06 | 0.17 |
| hIP8 | 49.8 ± 6.9 | -0.11 | 0.542 | -0.02 | 0.20 |

hIP1–hIP8 refer to human intraparietal areas 1–8, all defined in the left hemisphere. *: $p < 0.05$ (one-tailed), corrected for multiple comparisons across the eight ROIs; †: $p < 0.05$ (one-tailed), uncorrected.

Supplementary Table S7. MVPA results for 12-mm functional spherical ROIs ($n$ = 19) (primary ROI)

| ROI | Mean accuracy ± SD | t value | p value (one-tailed) | Effect size | $BF_{+0}$ |
|---|---|---|---|---|---|
| Execution task | | | | | |
| LS1 | 73.4 ± 11.2 | 8.88 | < 0.001* | 2.04 | 521300 |
| LM1 | 68.5 ± 10.8 | 7.24 | < 0.001* | 1.66 | 35940 |
| LPMd | 59.6 ± 10.4 | 3.92 | < 0.001* | 0.90 | 74.21 |
| LIPS | 59.1 ± 9.5 | 4.08 | < 0.001* | 0.94 | 101 |
| Language task | | | | | |
| LS1 | 49.9 ± 8.2 | -0.05 | 0.521 | -0.01 | 0.23 |
| LM1 | 50.4 ± 7.8 | 0.23 | 0.41 | 0.05 | 0.29 |
| LPMd | 52.3 ± 6.9 | 1.39 | 0.091 | 0.32 | 0.98 |
| LIPS | 54.5 ± 7.7 | 2.45 | 0.012* | 0.56 | 4.84 |
| Cross-classification (direction-averaged) | | | | | |
| LS1 | 51.2 ± 4.3 | 1.19 | 0.125 | 0.27 | 0.76 |
| LM1 | 50.2 ± 4.5 | 0.19 | 0.427 | 0.04 | 0.28 |
| LPMd | 50.9 ± 4.2 | 0.91 | 0.188 | 0.21 | 0.55 |
| LIPS | 51.9 ± 6.4 | 1.28 | 0.109 | 0.29 | 0.85 |
| Cross-classification (Execution → Language) | | | | | |
| LS1 | 49.3 ± 4.1 | -0.74 | 0.765 | -0.17 | 0.15 |
| LM1 | 49.1 ± 5.5 | -0.72 | 0.759 | -0.16 | 0.15 |
| LPMd | 50.4 ± 4.0 | 0.42 | 0.341 | 0.10 | 0.33 |
| LIPS | 50.8 ± 6.9 | 0.48 | 0.319 | 0.11 | 0.35 |
| Cross-classification (Language → Execution) | | | | | |
| LS1 | 52.6 ± 7.0 | 1.59 | 0.065 | 0.36 | 1.28 |
| LM1 | 51.5 ± 6.4 | 1.01 | 0.163 | 0.23 | 0.61 |
| LPMd | 51.5 ± 6.2 | 1.01 | 0.163 | 0.23 | 0.61 |
| LIPS | 53.0 ± 7.7 | 1.65 | 0.058 | 0.38 | 1.39 |

*: $p < 0.05$ (one-tailed), corrected for multiple comparisons across the four ROIs; †: $p < 0.05$ (one-tailed), uncorrected.

Supplementary Table S8. MVPA results for 10-mm functional spherical ROIs ($n$ = 19)

| ROI | Mean accuracy ± SD | t value | p value (one-tailed) | Effect size | $BF_{+0}$ |
|---|---|---|---|---|---|
| Execution task | | | | | |
| LS1 | 66.8 ± 11.1 | 6.44 | < .001* | 1.48 | 8910 |
| LM1 | 65.1 ± 13.6 | 4.71 | < .001* | 1.08 | 341 |
| LPMd | 58.0 ± 9.7 | 3.52 | 0.001* | 0.81 | 33.85 |
| LIPS | 57.6 ± 6.7 | 4.79 | < .001* | 1.10 | 396 |
| Language task | | | | | |
| LS1 | 52.0 ± 9.6 | 0.88 | 0.196 | 0.20 | 0.53 |
| LM1 | 52.7 ± 8.9 | 1.28 | 0.109 | 0.29 | 0.84 |
| LPMd | 50.9 ± 6.8 | 0.54 | 0.297 | 0.13 | 0.38 |
| LIPS | 54.2 ± 6.0 | 2.98 | 0.004* | 0.68 | 12.50 |
| Cross-classification (direction-averaged) | | | | | |
| LS1 | 51.4 ± 6.2 | 0.96 | 0.174 | 0.22 | 0.58 |
| LM1 | 50.8 ± 5.0 | 0.70 | 0.247 | 0.16 | 0.44 |
| LPMd | 50.6 ± 4.3 | 0.57 | 0.288 | 0.13 | 0.39 |
| LIPS | 53.8 ± 5.4 | 2.98 | 0.004* | 0.68 | 12.38 |
| Cross-classification (Execution → Language) | | | | | |
| LS1 | 50.3 ± 5.0 | 0.23 | 0.410 | 0.05 | 0.29 |
| LM1 | 49.3 ± 4.9 | -0.58 | 0.717 | -0.13 | 0.16 |
| LPMd | 52.0 ± 4.3 | 2.04 | 0.028† | 0.47 | 2.47 |
| LIPS | 52.7 ± 5.6 | 2.06 | 0.027† | 0.47 | 2.58 |
| Cross-classification (Language → Execution) | | | | | |
| LS1 | 52.2 ± 9.0 | 1.04 | 0.157 | 0.24 | 0.63 |
| LM1 | 53.0 ± 7.2 | 1.76 | 0.048† | 0.40 | 1.63 |
| LPMd | 50.3 ± 7.1 | 0.18 | 0.429 | 0.04 | 0.27 |
| LIPS | 54.1 ± 7.8 | 2.23 | 0.019† | 0.51 | 3.40 |

*: $p < 0.05$ (one-tailed), corrected for multiple comparisons across the four ROIs; †: $p < 0.05$ (one-tailed), uncorrected.

Supplementary Table S9. MVPA results for 15-mm functional spherical ROIs ($n$ = 19)

| ROI | Mean accuracy ± SD | t value | p value (one-tailed) | Effect size | $BF_{+0}$ |
|---|---|---|---|---|---|
| Execution task | | | | | |
| LS1 | 76.9 ± 10.0 | 11.39 | < 0.001* | 2.61 | $1.83 \times 10^{7}$ |
| LM1 | 73.0 ± 10.6 | 9.17 | < 0.001* | 2.10 | 810900 |
| LPMd | 64.0 ± 10.5 | 5.64 | < 0.001* | 1.29 | 2033 |
| LIPS | 60.7 ± 9.7 | 4.70 | < 0.001* | 1.08 | 334 |
| Language task | | | | | |
| LS1 | 52.8 ± 6.0 | 1.97 | 0.032† | 0.45 | 2.23 |
| LM1 | 50.4 ± 7.7 | 0.24 | 0.407 | 0.06 | 0.29 |
| LPMd | 50.6 ± 6.0 | 0.44 | 0.333 | 0.10 | 0.34 |
| LIPS | 54.5 ± 5.4 | 3.53 | 0.001* | 0.81 | 34.96 |
| Cross-classification (direction-averaged) | | | | | |
| LS1 | 50.5 ± 5.0 | 0.41 | 0.342 | 0.10 | 0.33 |
| LM1 | 51.0 ± 5.9 | 0.73 | 0.236 | 0.17 | 0.45 |
| LPMd | 51.1 ± 4.1 | 1.13 | 0.136 | 0.26 | 0.71 |
| LIPS | 51.7 ± 6.7 | 1.06 | 0.152 | 0.24 | 0.65 |
| Cross-classification (Execution → Language) | | | | | |
| LS1 | 50.2 ± 4.5 | 0.22 | 0.416 | 0.05 | 0.28 |
| LM1 | 48.8 ± 4.7 | -1.11 | 0.859 | -0.25 | 0.13 |
| LPMd | 50.6 ± 3.6 | 0.73 | 0.238 | 0.17 | 0.45 |
| LIPS | 51.3 ± 7.2 | 0.79 | 0.22 | 0.18 | 0.48 |
| Cross-classification (Language → Execution) | | | | | |
| LS1 | 50.8 ± 6.9 | 0.46 | 0.326 | 0.11 | 0.35 |
| LM1 | 52.7 ± 8.3 | 1.36 | 0.095 | 0.31 | 0.94 |
| LPMd | 52.2 ± 6.8 | 1.37 | 0.094 | 0.31 | 0.95 |
| LIPS | 52.8 ± 6.9 | 1.75 | 0.049† | 0.40 | 1.61 |

*: $p < 0.05$ (one-tailed), corrected for multiple comparisons across the four ROIs; †: $p < 0.05$ (one-tailed), uncorrected.

Supplementary Table S10. Effect of task-order on MVPA accuracy in 12-mm functional spherical ROIs (execution-first: *n* = 11; language-first: *n* = 13) (primary ROI)

| ROI | Order | Mean accuracy ± SD | t value | p value (two-tailed) | Effect size | $BF_{10}$ |
|---|---|---|---|---|---|---|
| Execution task | | | | | | |
| LS1 | Ex→La | 77.1 ± 12.4 | 1.09 | 0.286 | 0.45 | 0.58 |
| | La→Ex | 71.6 ± 11.1 | | | | |
| LM1 | Ex→La | 71.4 ± 12.4 | 0.88 | 0.390 | 0.36 | 0.50 |
| | La→Ex | 67.6 ± 7.7 | | | | |
| LPMd | Ex→La | 62.4 ± 10.5 | 0.48 | 0.639 | 0.20 | 0.41 |
| | La→Ex | 60.3 ± 9.8 | | | | |
| LIPS | Ex→La | 57.0 ± 5.3 | 0.76 | 0.456 | 0.31 | 0.46 |
| | La→Ex | 59.9 ± 11.1 | | | | |
| Language task | | | | | | |
| LS1 | Ex→La | 50.2 ± 7.7 | 0.43 | 0.670 | 0.18 | 0.40 |
| | La→Ex | 48.7 ± 8.3 | | | | |
| LM1 | Ex→La | 53.0 ± 7.2 | 1.91 | 0.069 | 0.78 | 1.34 |
| | La→Ex | 47.1 ± 7.2 | | | | |
| LPMd | Ex→La | 52.6 ± 7.3 | 0.28 | 0.781 | 0.12 | 0.39 |
| | La→Ex | 53.4 ± 6.0 | | | | |
| LIPS | Ex→La | 53.8 ± 7.0 | 0.04 | 0.971 | 0.02 | 0.37 |
| | La→Ex | 53.7 ± 7.7 | | | | |
| Cross-classification (direction-averaged) | | | | | | |
| LS1 | Ex→La | 51.0 ± 4.5 | 0.02 | 0.986 | 0.01 | 0.37 |
| | La→Ex | 51.0 ± 4.7 | | | | |
| LM1 | Ex→La | 51.0 ± 3.1 | 0.76 | 0.457 | 0.31 | 0.46 |
| | La→Ex | 49.7 ± 4.7 | | | | |
| LPMd | Ex→La | 52.9 ± 3.4 | 2.55 | 0.018† | 1.04 | 3.32 |
| | La→Ex | 49.0 ± 3.7 | | | | |
| LIPS | Ex→La | 51.7 ± 4.9 | 0.32 | 0.754 | 0.13 | 0.39 |
| | La→Ex | 50.9 ± 7.1 | | | | |

Participants were divided according to task order (execution-first vs. language-first). Two-sample t-tests were conducted for each ROI.

*: $p < 0.05$ (two-tailed), corrected for multiple comparisons across the four ROIs; †: $p < 0.05$ (two-tailed), uncorrected.

Supplementary Table S11. Effect of task-order on MVPA accuracy in 10-mm functional spherical ROIs (execution-first: $n$ = 11; language-first: $n$ = 13)

| ROI | Order | Mean accuracy ± SD | t value | p value (two-tailed) | Effect size | $BF_{10}$ |
|---|---|---|---|---|---|---|
| Execution task | | | | | | |
| LS1 | Ex→La | 70.1 ± 10.0 | 1.09 | 0.288 | 0.45 | 0.57 |
| | La→Ex | 65.2 ± 10.9 | | | | |
| LM1 | Ex→La | 70.3 ± 12.6 | 1.42 | 0.171 | 0.58 | 0.76 |
| | La→Ex | 63.0 ± 11.6 | | | | |
| LPMd | Ex→La | 56.0 ± 10.3 | 0.89 | 0.384 | 0.36 | 0.50 |
| | La→Ex | 59.9 ± 10.5 | | | | |
| LIPS | Ex→La | 55.9 ± 5.4 | 1.08 | 0.291 | 0.44 | 0.57 |
| | La→Ex | 58.8 ± 7.0 | | | | |
| Language task | | | | | | |
| LS1 | Ex→La | 50.9 ± 8.5 | 0.14 | 0.889 | 0.06 | 0.38 |
| | La→Ex | 51.4 ± 9.5 | | | | |
| LM1 | Ex→La | 53.7 ± 7.6 | 0.93 | 0.360 | 0.38 | 0.51 |
| | La→Ex | 50.5 ± 8.6 | | | | |
| LPMd | Ex→La | 49.1 ± 7.1 | 1.31 | 0.205 | 0.54 | 0.69 |
| | La→Ex | 53.5 ± 8.3 | | | | |
| LIPS | Ex→La | 55.8 ± 6.3 | 1.94 | 0.065 | 0.80 | 1.39 |
| | La→Ex | 51.0 ± 5.1 | | | | |
| Cross-classification (direction-averaged) | | | | | | |
| LS1 | Ex→La | 51.7 ± 5.7 | 0.02 | 0.988 | 0.01 | 0.37 |
| | La→Ex | 51.8 ± 6.4 | | | | |
| LM1 | Ex→La | 49.3 ± 4.5 | 1.32 | 0.200 | 0.54 | 0.70 |
| | La→Ex | 51.7 ± 4.3 | | | | |
| LPMd | Ex→La | 50.4 ± 2.9 | 0.46 | 0.648 | 0.19 | 0.41 |
| | La→Ex | 51.2 ± 4.7 | | | | |
| LIPS | Ex→La | 55.1 ± 4.4 | 1.53 | 0.140 | 0.63 | 0.86 |
| | La→Ex | 51.9 ± 5.2 | | | | |

Participants were divided according to task order (execution-first vs. language-first). Two-sample t-tests were conducted for each ROI.

*: $p < 0.05$ (two-tailed), corrected for multiple comparisons across the four ROIs; †: $p < 0.05$ (two-tailed), uncorrected.

Supplementary Table S12. Effect of task-order on MVPA accuracy in 15-mm functional spherical ROIs (execution-first: $n$ = 11; language-first: $n$ = 13)

| ROI | Order | Mean accuracy ± SD | t value | p value (two-tailed) | Effect size | $BF_{10}$ |
|---|---|---|---|---|---|---|
| Execution task | | | | | | |
| LS1 | Ex→La | 80.8 ± 9.5 | 1.42 | 0.169 | 0.58 | 0.77 |
| | La→Ex | 74.8 ± 10.1 | | | | |
| LM1 | Ex→La | 76.2 ± 11.5 | 1.11 | 0.280 | 0.45 | 0.58 |
| | La→Ex | 71.4 ± 9.2 | | | | |
| LPMd | Ex→La | 67.7 ± 9.0 | 0.83 | 0.413 | 0.34 | 0.48 |
| | La→Ex | 63.9 ± 11.5 | | | | |
| LIPS | Ex→La | 58.3 ± 7.9 | 1.21 | 0.241 | 0.49 | 0.63 |
| | La→Ex | 62.7 ± 9.3 | | | | |
| Language task | | | | | | |
| LS1 | Ex→La | 52.3 ± 8.9 | 0.50 | 0.625 | 0.20 | 0.41 |
| | La→Ex | 50.8 ± 5.4 | | | | |
| LM1 | Ex→La | 50.9 ± 7.8 | 0.63 | 0.535 | 0.26 | 0.43 |
| | La→Ex | 48.9 ± 7.0 | | | | |
| LPMd | Ex→La | 50.7 ± 5.7 | 0.25 | 0.809 | 0.10 | 0.38 |
| | La→Ex | 50.0 ± 6.8 | | | | |
| LIPS | Ex→La | 52.9 ± 5.2 | 1.54 | 0.137 | 0.63 | 0.87 |
| | La→Ex | 56.6 ± 5.8 | | | | |
| Cross-classification (direction-averaged) | | | | | | |
| LS1 | Ex→La | 50.8 ± 3.8 | 0.40 | 0.693 | 0.16 | 0.40 |
| | La→Ex | 50.0 ± 5.5 | | | | |
| LM1 | Ex→La | 51.7 ± 2.3 | 0.50 | 0.621 | 0.21 | 0.41 |
| | La→Ex | 50.6 ± 6.9 | | | | |
| LPMd | Ex→La | 51.2 ± 4.8 | 0.49 | 0.626 | 0.20 | 0.41 |
| | La→Ex | 50.4 ± 2.3 | | | | |
| LIPS | Ex→La | 52.0 ± 6.4 | 0.39 | 0.697 | 0.16 | 0.40 |
| | La→Ex | 50.9 ± 6.1 | | | | |

Participants were divided according to task order (execution-first vs. language-first). Two-sample t-tests were conducted for each ROI.

*: $p < 0.05$ (two-tailed), corrected for multiple comparisons across the four ROIs; †: $p < 0.05$ (two-tailed), uncorrected.
